# Association between the American Heart Association’s New Life’s Essential 8 Metrics and Depression Symptom in U.S General Adults, Finding from NHANES 2005-2018

**DOI:** 10.3389/fpsyt.2024.1480036

**Published:** 2024-11-18

**Authors:** Zhuoer Ruan, Jie Zhu, Shengnan Xu, Jinghong Liang, Shengqiao Shi

**Affiliations:** ^1^ Department of Psychiatry, The Second Affiliated Hospital and Yuying Children’s Hospital of Wenzhou Medical University, Wenzhou, China; ^2^ Department of Maternal and Child Health, School of Public Health, Sun Yat-sen University, Guangzhou, China; ^3^ Department of Respiratory Medicine, The Second Affiliated Hospital and Yuying Children’s Hospital of Wenzhou Medical University, Wenzhou, China

**Keywords:** life’s essential 8, cardiovascular health, depression symptom, general adults, cross-sectional study

## Abstract

**Background:**

The American Heart Association (AHA) recently introduced a new metric for promoting cardiovascular health (CVH) called Life’s Essential 8 (LE8). However, there has been no investigation into the relationship between levels of LE8 and the risk of depression symptom. Therefore, our objective was to determine this association using a nationally representative sample of U.S adults.

**Methods:**

Utilizing cross-sectional data from the NHANES spanning the years 2005 to 2018, we computed scores for both overall CVH and individual LE8 components. The survey-weighted logistic regression models were conducted to determine whether LE8 was associated with depression symptom.

**Results:**

A total of 25,357 adults aged 20 and above were included in the study, representing a population of 1,184 million non-institutionalized U.S residents. The study revealed that individuals with positive scores in both individual and total LE8 metrics were less likely to experience depressive symptoms compared to those with negative scores. Furthermore, a significant negative linear trend was observed, showing that as the overall number of favorable LE8 scores increased, the likelihood of depressive symptoms decreased.

**Conclusion:**

Attaining a higher CVH score, as defined by the LE8, is strongly linked to a lower risk of experiencing depressive symptoms in adult residents of the U.S.

## Introduction

Depression is a common mental disorder that impacts a significant number of individuals globally. Approximately 280 million people are affected by depression, contributing to over 47 million disability-adjusted life years in 2019 (1). High-income countries bear a considerable disease burden related to depression, making it the second leading cause of disability-adjusted life years and leading to a notable decrease in productivity (2–5).

Depressive disorders can potentially be prevented through effective interventions that focus on modifying established risk factors (6, 7). Current studies have not placed enough emphasis on identifying effective prevention methods for depression (8). Therefore, it is crucial to identify modifiable risk factors associated with depression to develop effective prevention strategies. The ‘vascular depression’ hypothesis suggests that cardiovascular factors play a significant role as a risk factor for depression (9). Ideal cardiovascular health (ICH) is a tool developed by the American Heart Association (AHA) to assess and track cardiovascular health (CVH) using the Life’s Simple 7 (LS7) framework. This framework is based on four health behaviors: physical activity (PA), nonsmoking, maintaining a healthy body weight, and following an optimal diet, along with three biological metrics: untreated blood glucose, total cholesterol (TC), and blood pressure at optimal levels. Previous studies have shown that LS7 can effectively prevent depression (10, 11). However, the association between LS7 and depression symptoms in prior research has been inconsistent, with one study reporting a significant negative relationship (12) and another finding an insignificant one (9). Previous studies have been limited by small sample sizes, often including only thousands of participants or fewer, and focusing specifically on the elderly (11). The LS7 framework, used in previous research, has its own limitations such as concentrating solely on certain health behaviors without considering sleep health, and utilizing a basic scoring system that may not capture individual variances or changes over time (13). In response to these limitations, the AHA has introduced the LE8 metrics to address some of the shortcomings of the LS7 framework (13). These recent updates have brought about several enhancements, such as offering a more comprehensive explanation of score categories, incorporating sleep health as an additional metric, broadening the definition of a nutritious diet, and including hemoglobin A1c (HbA1c) levels in conjunction with fasting blood glucose (FBG). Regrettably, there is a lack of research assessing the efficacy of LE8’s cardiovascular health metrics in relation to depression symptoms within a diverse general population. The Black-White mental health paradox (14) highlights disparities in mental health conditions across various genders, races, and ethnicities. Previous research has not thoroughly investigated the influence of LE8 on depression and its potential role in gender and racial/ethnic inequalities. Furthermore, several studies have overlooked important risk factors for depression, including alcohol consumption and family income (15, 16). Over the past decade, researchers have increasingly acknowledged the significant association between depression and cardiovascular disease. However, a comprehensive understanding of this comorbidity remains limited, particularly regarding pathophysiological mechanisms. For instance, inflammation is considered a crucial factor linking these two conditions (17). Inflammation not only plays a central role in the progression of cardiovascular disease but is also strongly correlated with the severity of depressive symptoms. Furthermore, imbalances in the autonomic nervous system have been identified as a potential mechanism for the co-occurrence of depression and cardiovascular disease (18). Such imbalances can result in abnormal heart function and worsen mood disorders. Metabolic syndrome, encompassing obesity, hypertension, and insulin resistance, has also been implicated in the pathogenesis of both conditions (19). Exploring the potential link between LE8 and depression symptoms could offer valuable insights into preventing depression by promoting a combination of healthy behaviors.

Utilizing data from the National Health and Nutrition Examination Survey (NHANES) conducted between 2005 and 2018, this study aimed to investigate the correlation between cardiovascular health (CVH) levels, as assessed by the LE8 score, and the likelihood of experiencing depressive symptoms among U.S. adults. Additionally, we explored these relationships by stratifying the data according to gender and race/ethnicity, considering the established disparities in life expectancy within these demographic groups.

## Methods

### Participants and study design

This study utilized data from the National Health and Nutrition Examination Survey (NHANES), adhering to all relevant ethical guidelines. The NHANES study received approval from the National Center for Health Statistics (NCHS) of the Centers for Disease Control and Prevention (CDC). Research Ethics Review Board (ERB) under IRB protocol number 2021-05 for the 2021-2022 cycle. All participants in the NHANES study provided written informed consent. It uses a complex, multistage, stratified, clustered probability sampling design to assess the health and nutritional status of the U.S civilian non-institutionalized population. Eligible participants were invited to participate in in-home interviews, where information on demographic, socioeconomic, lifestyle, and other health-related questions was obtained. Additionally, physical examinations were conducted, which included anthropometric and biological measurements. A comprehensive description of the study design and methodology of NHANES has been provided in previous literature (https://www.cdc.gov/nchs/nhanes/about_nhanes.htm).

**Figure 1:**
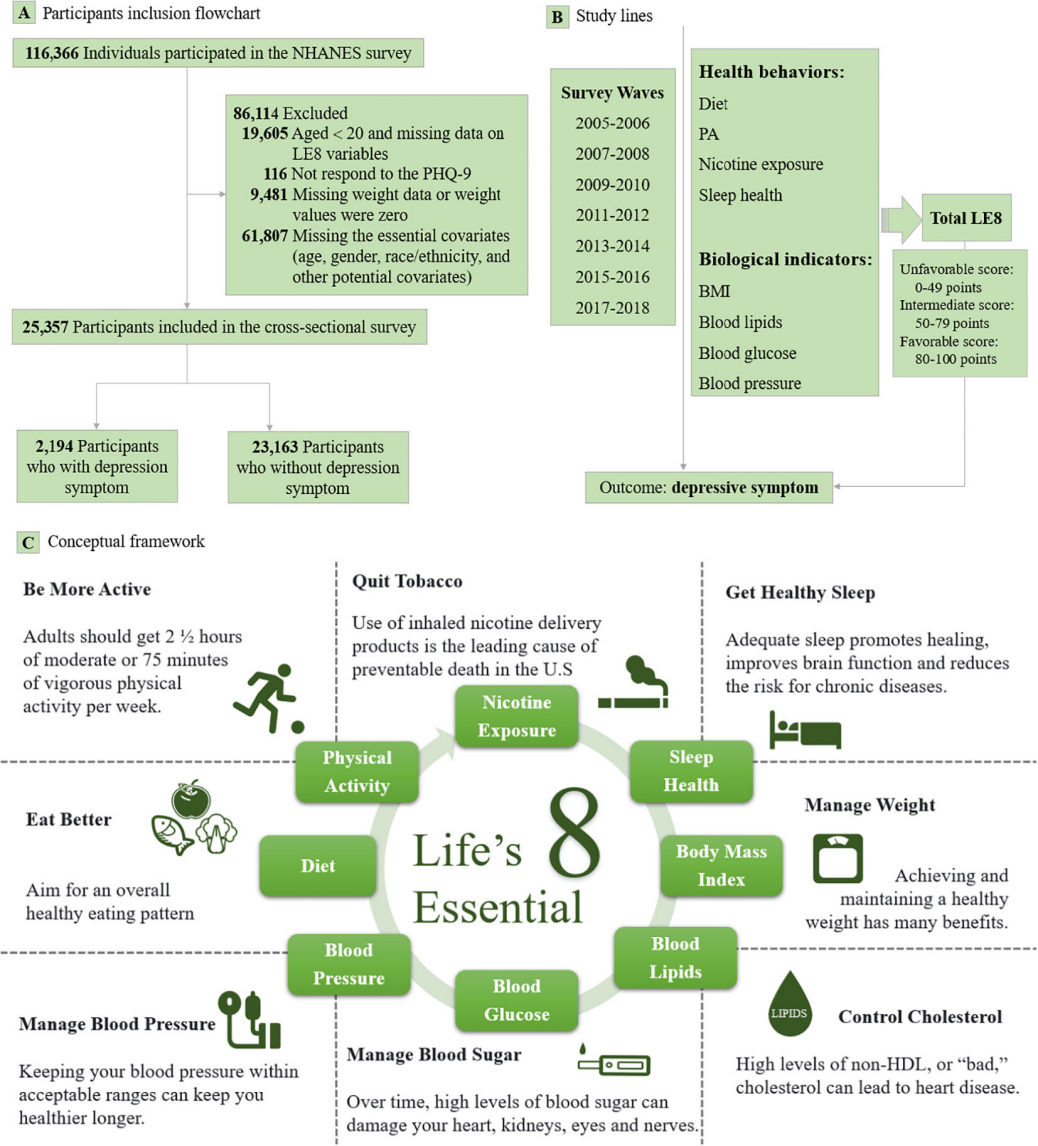
Flowchart of selection of NHANES participants along with study lines and conceptual framework. AHA, American Heart Association; BMI, Body mass undex; LE8, Life’s Essential 8; NHANES, National Health and Nutrition Examination Survey; PA, Physical activity; PHQ-9, Patient health questionnaire-9. Lables **(A–C)** are Participants inclusion flowchart, Study lines and Conceptual framework, respectively.

This study utilized cross-sectional data from seven consecutive nonrepetitive survey waves of the NHANES database (2005−2006, 2007−2008, 2009−2010, 2011−2012, 2013−2014, 2015−2016, 2017−2018). Initially, a total of 116,366 participants were included in this study. We excluded individuals aged less than 20 years and those with missing data on LE8 variables (n=19,605), and those who did not respond to the patient health questionnaire-9 (PHQ-9) (n=116). Furthermore, we excluded individuals with missing or zero weight values (n=9,481), as well as those who lacked information on essential covariates such as age, gender, race/ethnicity, and other potential covariates (n=61,807). Ultimately, a total of 25,357 participants were eligible for this analysis (**Figure 1**).

### Measurements of LE8

The LE8 is a quantitative metric of CVH, that comprises two domains: health behaviors including diet, PA, nicotine exposure, and sleep health, as well as biological indicators such as body mass index (BMI), blood lipids, blood glucose, and blood pressure. Diet quality was assessed based on the Healthy Eating Index 2015. The mean values of each dietary component collected from 2 interviewer-administered nonconsecutive 24-hour dietary recalls were used for the assessment (20). Information on PA was collected through self-reporting frequency and duration of moderate or vigorous PA per week using standardized questionnaires. The exposure to nicotine was assessed based on self-reported usage of combustible tobacco or inhalation nicotine delivery systems (i.e., vaping devices, e-cigarettes, and secondhand tobacco smoke). In LE8, the metric for sleep health was assessed through self-reported average hours of sleep per night using standardized questionnaires. BMI was determined by dividing weight in kilograms (kg) by standing height in meters squared(m^2^). Blood samples were collected for the assessment of blood lipids, fasting glucose, and HbA1c. The concentration of non-high density lipoprotein (non-HDL) cholesterol was calculated by subtracting high-density lipoprotein (HDL) cholesterol from TC. FBG was measured based on fasting blood samples. HbA1c levels were determined using high-performance liquid chromatography, which involved analyzing both fasting and non-fasting blood samples. Blood pressure was measured using an Omron device to estimate systolic and diastolic blood pressure by calculating the average of available blood pressure measurements.

Weak correlations were observed between the eight items of LE8 (**Supplementary Table S1**). **Supplementary Table S2** provided detailed information on eight health and biological items, as well as the scoring algorithm for each LE8 metric. The total LE8 score was calculated by averaging the sum of scores from all eight items, resulting in a total score ranging from 0 to 100. In this study, three categories were coded to estimate the levels of CVH based on individual and total LE8 scores: an unfavorable score (0-49 points), an intermediate score (50-79 points), and a favorable score (80-100 points) following the recommendation by AHA (13). In order to enhance statistical power, we standardized the score to 800 for calculating RCS to elucidate the detailed linear relationship between LE8 and depression symptom.

### Assessments on depression symptom

The PHQ-9 (21), a widely accepted questionnaire known for its established validity and reliability (22), was used to identify depression symptom in NHANES participants over the course of the last days during each survey wave. Participants completed a PHQ-9 questionnaire, rating each of the nine items on a scale of 0 to 3. This resulted in a total score ranging from 0 to 27, with a higher score indicating more severe depressive symptom. Participants who score 10 or higher were identified as having depression symptom. The initial categorization consisted of five tiers: 0–4 points (none to minimal), 5–9 points (mild), 10–14 points (moderate), 15–19 points (moderately severe), and 20–27 points (severe).

### Assessment of covariates

Multiple covariates were evaluated as potential confounders according to previous studies (23–25). Information on sociodemographic characteristics was obtained through standardized questionnaires. These covariates presented in this study included age (20-39, 40-59, 60-79, ≥ 80 years), gender (male, female), race/ethnicity [Mexican American, non-Hispanic White, non-Hispanic Black, other races (including multiracial and other Hispanic)], family poverty-to-income ratio (PIR) [(≥ 300%, < 300%), The PIR is a measurement used to classify household income in relation to the federal poverty line. Families with a PIR value less than 1.0 are considered to be living below the federal poverty line], alcohol consumption (never, former, current), education level, and marital status. Educational levels were categorized into five groups: less than 9th grade, 9th-11th grade (including 12th grade with no diploma), high school grade/general equivalent diploma (GED) or equivalent, some college or associate’s degree (AA) degree, and college graduate or above. Marital status was divided into three categories: married/living with a partner, never married, and widowed/divorced/separated.

### Statistical analyses

In our study, we considered the intricate sampling design and weights, and rigorously validated our results. Adhering to the NHANES Analytic and Reporting Guidelines, we applied sample weights that addressed non-response and non-coverage disparities, along with adjustments for oversampling particular demographics (26). To guarantee the national population’s representation, we recalibrated the weights utilizing the 2-year MEC weight divided by 6, spanning six consecutive NHANES waves, as per NHANES recommendations (https://wwwn.cdc.gov/nchs/nhanes/tutorials/weighting.aspx) (27). Continuous variables following a normal distribution were expressed as means ± standard error (SE) while categorical variables were reported as counting (n) and survey-weighted percentage (%). Weighted Chi-square tests were used for categorical variables, ANOVA analyses and *t*-tests were used for examining continuous variables. Pairwise correlations were conducted to identify potential multicollinearity among the eight LE8 items. Descriptive analysis was used to examine the participants based on the three categories of total LE8 scores. Survey-weighted logistic regression models were conducted to assess the association between different levels of individual and total LE8 scores and depression symptom. This was done by estimating the adjusted odds ratio (AOR) and corresponding 95% confidence intervals (CIs). In our main analyses, three models were established using the unfavorable score as a reference. The crude model, which was unadjusted, was applied to each sub-item of the LE8 score. Model 1 was adjusted for age, gender, and race/ethnicity. Model 2 was adjusted for age, gender, race/ethnicity, PIR, education level, alcohol consumption, marital status, and the other 7 items of LE8. In the case of total LE8 score, Model 2 was adjusted for age, gender, race/ethnicity, PIR, education level, alcohol consumption, and marital status.

Stratified analyses were performed based on several variables (gender, race/ethnicity, and survey waves), and the trend tests were also applied in these analyses. A battery of sensitivity analyses was conducted to further investigate the results: Firstly, on the basis of Model 2, we further adjusted for survey waves, diabetes mellitus (DM), hypertension, cardiovascular diseases (CVDs), total energy intake, whether the participants were using an antidepressant, as well as the combination of DM, hypertension, and CVDs, respectively. To assess the relevant associations, we divided the participants into two groups based on the median value of individual and total LE8 scores. The groups were divided as follows: one group consisted of participants with scores less than the median, while the other group consisted of participants with scores greater than or equal to the median. Thirdly, we evaluated the individual and total LE8 scores associated with depressive symptom by using the favorable score as the reference. Finally, to assess the cumulative effect, we calculated the total number of favorable LE8 components. We also established binary categorical variables (unfavorable/intermediate score, favorable score) for each of the eight LE8 items to evaluate the robustness of our main analyses. R language was used for all statistical analyses in this study. All statistical tests were two-sided, *P* < 0.05 was defined as statistically significant.

## Results

### Characteristics of study participants

A total of 25,357 NHANES participants aged 20 years or older with valid depression symptom outcomes were included in seven survey waves conducted between 2005 and 2018, representing 1,184 million non-institutional U.S residents with 49.0% being male (n=12,431). Out of all the participants, the age-adjusted prevalence of depression symptom was 8.7% (n=2,194), and the weighted mean age of all participants was 47.98 (0.26) years. The largest proportion of participants identified as non-Hispanic White (46.1%, n=11,681), followed by non-Hispanic Black (21.1%, n=5,337), other races (18.4%, n=4,665), and Mexican American (14.5%, n=3,674). Significant differences were observed among participants with different levels of total LE8 score (unfavorable score, intermediate score, and favorable score) in terms of age, gender, and other demographic variables. **Table 1** presented the survey-weighted characteristics of the study participants classified into three levels based on their total LE8 scores. **Supplementary Tables S3**-**S6** present demographic characteristics and pertinent epidemiological data for the participants categorized by depression symptoms, gender, race/ethnicity, and survey waves.

**Table 1:** Survey-weighted characteristic variables of the study participants stratified by three levels of total LE8 score, NHANES 2005-2018, U.S (n = 25,357).

Characteristic variables	Age-adjusted prevalence rate^a^	Estimate U.S population (n)	Total participants [n (%)]	Total LE8 score			*P*-value^b^
				Unfavorable score (0-49 points)	Intermediate score (50-79 points)	Favorable score (80-100 points)	
**No. of participants**	7.9 (0.3)	1,184,141,254	25,357 (100.0)	4,043 (15.9)	16,765 (66.1)	4,549 (17.9)	-
**Age, years**	-	-	47.98 ± 0.26	53.34 ± 0.37	48.90 ± 0.28	41.98 ± 0.43	**< 0.001**
20-39	7.9 (0.4)	408,025,337	8,203 (32.4)	678 (20.1)	5,150 (32.2)	2,375 (49.9)	**< 0.001**
40-59	9.3 (0.5)	459,397,489	8,571 (33.8)	1,524 (43.8)	5,683 (39.4)	1,364 (34.1)	
60-79	6.2 (0.5)	271,522,095	7,142 (28.2)	1,587 (31.3)	4,874 (24.1)	681 (14.3)	
≥ 80	4.1 (0.6)	45,196,334	1,441 (5.7)	254 (4.9)	1,058 (4.3)	129 (1.7)	
Gender							
Male	6.2 (0.4)	571,113,588	12,431 (49.0)	1,962 (47.6)	8,644 (50.8)	1,825 (40.8)	**< 0.001**
Female	9.6 (0.4)	613,027,666	12,926 (51.0)	2,081 (52.5)	8,121 (49.2)	2,724 (59.2)	
Race/ethnicity							
Mexican American	8.6 (0.7)	89,101,932	3,674 (14.5)	525 (7.0)	2,535 (7.9)	614 (6.8)	**< 0.001**
Non-Hispanic Black	9.8 (0.5)	124,176,625	5,337 (21.1)	1,252 (17.8)	3,534 (10.7)	551 (5.6)	
Non-Hispanic White	7.4 (0.4)	833,102,497	11,681 (46.1)	1,728 (65.0)	7,737 (70.2)	2,216 (73.9)	
Other races	9.8 (0.8)	137,760,201	4,665 (18.4)	538 (10.2)	2,959 (11.2)	1,168 (13.7)	
**BMI, kg/m^2^**	-	-	29.12 ± 0.09	34.76 ± 0.21	29.53 ± 0.08	24.50 ± 0.09	**< 0.001**
**PIR**	-	-	3.07 ± 0.04	2.41 ± 0.05	3.05 ± 0.04	3.52 ± 0.05	**< 0.001**
≥ 300%	4.2 (0.3)	611,632,294	9,838 (38.8)	990 (33.8)	6,446 (51.0)	2,402 (64.3)	**< 0.001**
< 300%	12.4 (0.5)	572,508,960	15,519 (61.2)	3,053 (66.2)	10,319 (49.0)	2,147 (35.7)	
Education level							
Less than 9th grade	13.2 (1.3)	48,957,898	2,165 (8.5)	508 (7.6)	1,453 (4.1)	204 (2.1)	**< 0.001**
9-11th grade (including 12th grade with no diploma)	13.4 (0.9)	114,617,982	3,384 (13.4)	837 (17.8)	2,251 (9.9)	296 (4.2)	
High school grade/GED or equivalent	9.7 (0.6)	275,421,825	5,833 (23.0)	1,120 (30.5)	4,099 (25.6)	614 (11.9)	
Some college or AA degree	8.6 (0.5)	380,180,823	7,733 (30.5)	1,155 (31.4)	5,256 (33.5)	1,322 (28.2)	
College graduate or above	3.6 (0.4)	364,962,726	6,242 (24.6)	423 (12.7)	3,706 (26.9)	2,113 (53.6)	
Alcohol consumption							
Never	6.5 (0.6)	122,416,118	3,367 (13.3)	497 (9.8)	2,177 (10.0)	693 (11.7)	**< 0.001**
Former	11.1 (0.9)	160,448,474	4,239 (16.7)	1,075 (23.5)	2,786 (13.8)	378 (6.9)	
Current	7.5 (0.3)	901,276,662	17,751 (70.0)	2,471 (66.8)	11,802 (76.2)	3,478 (81.4)	
Marital status							
Married/Living with partner	5.9 (0.3)	752,598,329	15,371 (60.6)	2,219 (58.2)	10,319 (64.2)	2,833 (64.8)	**< 0.001**
Never married	10.4 (0.8)	209,613,147	4,420 (17.4)	554 (14.1)	2,692 (16.0)	1,174 (24.9)	
Widowed/Divorced/Separated	14.9 (0.9)	221,929,779	5,566 (22.0)	1,270 (27.8)	3,754 (19.7)	542 (10.3)	
Items of LE8 score (0-100 points)							
**Diet**	-	-	39.44 ± 0.51	21.65 ± 0.54	36.26 ± 0.48	59.75 ± 0.71	**< 0.001**
Unfavorable score	10.1 (0.5)	599,051,916	12,782 (50.4)	2,976 (76.1)	8,767 (54.6)	1,039 (23.2)	**< 0.001**
Intermediate score	6.3 (0.4)	289,423,345	6,302 (24.9)	747 (17.0)	4,313 (25.2)	1,242 (26.6)	
Favorable score	5.0 (0.4)	295,665,993	6,273 (24.7)	320 (6.9)	3,685 (20.2)	2,268 (50.2)	
**PA**	-	-	71.54 ± 0.50	30.88 ± 1.10	72.08 ± 0.53	94.34 ± 0.35	**< 0.001**
Unfavorable score	12.3 (0.6)	324,089,678	8,083 (31.9)	2,846 (69.7)	5,021 (26.6)	216 (4.2)	
Intermediate score	7.2 (1.2)	59,532,580	1,206 (4.8)	165 (4.5)	883 (5.8)	158 (2.9)	
Favorable score	6.3 (0.3)	800,518,996	16,068 (63.4)	1,032 (25.8)	10,861 (67.6)	4,175 (92.9)	**< 0.001**
**Nicotine exposure**	-	-	71.10 ± 0.49	44.52 ± 1.00	69.35 ± 0.50	92.36 ± 0.43	**< 0.001**
Unfavorable score	14.7 (0.7)	265,696,864	5,779 (22.8)	1,862 (48.9)	3,792 (23.7)	125 (2.7)	**< 0.001**
Intermediate score	6.5 (0.6)	268,062,407	5,707 (22.5)	988 (23.9)	4,043 (24.2)	676 (17.0)	
Favorable score	5.4 (0.3)	650,381,983	13,871 (54.7)	1,193 (27.2)	8,930 (52.1)	3,748 (80.3)	
**Sleep health**	-	-	83.35 ± 0.29	67.82 ± 0.73	83.36 ± 0.28	92.52 ± 0.36	**< 0.001**
Unfavorable score	19.0 (0.9)	175,478,530	4,467 (17.6)	1,539 (35.9)	2,730 (14.4)	198 (3.4)	**< 0.001**
Intermediate score	7.5 (0.6)	241,448,635	5,456 (21.5)	939 (23.1)	3,799 (21.6)	718 (15.3)	
Favorable score	5.6 (0.3)	767,214,089	15,434 (60.9)	1,565 (41.1)	10,236 (64.0)	3,633 (81.3)	
**BMI**	-	-	60.08 ± 0.45	33.10 ± 0.74	57.04 ± 0.41	85.45 ± 0.46	**< 0.001**
Unfavorable score	10.1 (0.4)	448,070,779	9,935 (39.2)	2,866 (73.4)	6,734 (41.1)	335 (6.8)	**< 0.001**
Intermediate score	6.5 (0.5)	388,661,523	8,341 (32.9)	825 (19.0)	6,043 (35.9)	1,473 (32.0)	
Favorable score	6.7 (0.5)	347,408,952	7,081 (27.9)	352 (7.6)	3,988 (23.1)	2,741 (61.3)	
**Blood lipids (non–HDL-C)**	-	-	63.49 ± 0.35	42.65 ± 0.77	60.63 ± 0.41	82.85 ± 0.56	**< 0.001**
Unfavorable score	8.8 (0.4)	440,283,865	9,330 (36.8)	2,636 (67.8)	6,211 (39.7)	483 (11.1)	**< 0.001**
Intermediate score	6.9 (0.4)	270,081,136	5,613 (22.1)	590 (14.6)	4,074 (24.8)	949 (21.8)	
Favorable score	7.7 (0.4)	473,776,253	10,414 (41.1)	817 (17.7)	6,480 (35.5)	3,117 (67.1)	
**Blood glucose**	-	-	86.05 ± 0.25	61.70 ± 0.68	86.07 ± 0.27	97.78 ± 0.20	**< 0.001**
Unfavorable score	11.7 (0.8)	166,598,615	4,671 (18.4)	2,078 (47.1)	2,554 (11.9)	39 (0.7)	**< 0.001**
Intermediate score	8.9 (0.8)	201,806,038	5,019 (19.8)	1,084 (27.8)	3,675 (19.1)	260 (4.4)	
Favorable score	7.0 (0.4)	815,736,601	15,667 (61.8)	881 (25.1)	10,536 (69.0)	4,250 (94.8)	
**Blood pressure**	-	-	69.03 ± 0.33	46.57 ± 0.67	66.52 ± 0.36	89.49 ± 0.42	**< 0.001**
Unfavorable score	9.1 (0.7)	249,775,713	6,307 (24.9)	2,045 (47.6)	4,105 (21.9)	157 (2.7)	**< 0.001**
Intermediate score	7.3 (0.5)	387,760,307	8,143 (32.1)	1,211 (31.7)	5,974 (36.8)	958 (21.0)	
Favorable score	7.8 (0.4)	546,605,234	10,907 (43.0)	787 (20.8)	6,686 (41.2)	3,434 (76.3)	
Survey waves							
2005-2006	5.9 (0.7)	170,066,111	3,388 (13.4)	546 (15.1)	2,414 (15.7)	428 (10.0)	**< 0.05**
2007-2008	8.6 (0.9)	160,988,009	3,964 (15.6)	677 (14.5)	2,637 (13.9)	650 (12.2)	
2009-2010	8.0 (0.6)	165,180,398	4,150 (16.4)	695 (14.0)	2,718 (13.7)	737 (14.6)	
2011-2012	8.2 (0.9)	176,400,748	3,625 (14.3)	579 (14.8)	2,309 (14.6)	737 (16.0)	
2013-2014	9.1 (0.7)	184,101,388	3,916 (15.4)	573 (15.8)	2,503 (14.8)	840 (17.6)	
2015-2016	7.8 (0.8)	174,615,296	3,541 (14.0)	553 (13.8)	2,345 (14.5)	643 (16.0)	
2017-2018	7.7 (0.7)	152,789,305	2,773 (10.9)	420 (12.0)	1,839 (12.9)	514 (13.5)	
Depressive symptom							
No	-	1,090,357,765	23,163 (91.4)	3,326 (81.6)	15,451 (92.5)	4,386 (97.1)	**< 0.001**
Yes	-	93,783,490	2,194 (8.7)	717 (18.4)	1,314 (7.5)	163 (2.9)	

Continuous variables are presented as weighted mean ± SE, and categorical variables are presented as counting (n) and survey-weighted percentage (%). The LE8 is a quantitative metric of CVH, that contains 8 items: diet, PA, nicotine exposure, sleep health, BMI, blood lipids (non–HDL-C), blood glucose, and blood pressure. The total LE8 score was calculated as the mean of the sum of all 8 items of LE8 score and similarly ranged from 0 (if the mean score of all items was 0) to 100 (optimal CVH). Score for total LE8 and its items was categorized into unfavorable score (0-49 points), intermediate score (50-79 points), and favorable score (80-100 points) according to AHA recommendation (13).

a Age-adjusted prevalence rates are present as [weighted number, % (SE)]. There were two steps to calculate them: First, the standard age proportions for age groups, based on the 2000 U.S Census Standard Population data, were calculated by dividing the age-specific Census population (P) by the total Census population number (T), and the standardizing proportions (P/T) should sum to 1. Second, the age-specific prevalence from the study population is multiplied by the proportion of people in that age group in the standard population, and results summed up to get the age-adjusted estimates. More detail can be got from: https://wwwn.cdc.gov/nchs/nhanes/tutorials/samplecode.aspx.

b The P-values were assessed by one-way ANOVA (continuous variables) or by chi-square test (categorical variables) to represent the differences among three levels of total CVH score. P-values presented with bold valued were statistically significant.

### Relationship between LE8 scores and depression symptom

**Table 2** identified the relationships between various levels of individual and total LE8 scores and the risk of depression symptom, while accounting for different covariates. In the fully adjusted model (Model 2), participants with intermediate scores for PA (AOR=0.681, 95% CI: 0.528, 0.869), nicotine exposure (AOR=0.645, 95% CI: 0.558, 0.744), and sleep health (AOR=0.506, 95% CI: 0.442, 0.579), along with favorable scores for diet (AOR=0.756, 95% CI: 0.656, 0.870), PA (AOR= 0.700, 95% CI: 0.631, 0.778), BMI (AOR= 0.722, 95% CI: 0.634, 0.821), and blood glucose (AOR= 0.672, 95% CI: 0.587, 0.770), were found to have a lower likelihood of experiencing depression symptom compared to those with unfavorable scores (**Table 2**).

**Table 2:** Comparison between different survey-weighted logistic regression models of the weighted relationship between total and individual LE8 scores and risk of depression symptom, NHANES 2005-2018, U.S (n = 25,357).

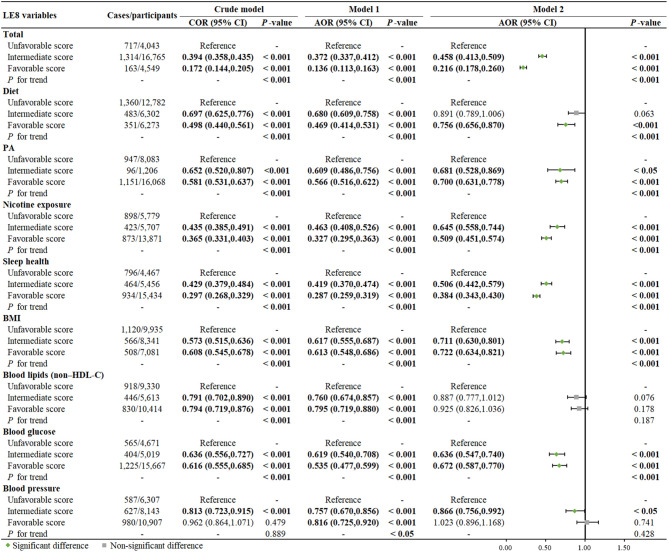

The LE8 is a quantitative metric of CVH, that contains 8 items: diet, PA, nicotine exposure, sleep health, BMI, blood lipids (non–HDL-C), blood glucose, and blood pressure. The total LE8 score was calculated as the mean of the sum of all 8 items of LE8 score and similarly ranged from 0 (if the mean score of all items was 0) to 100 (optimal CVH). Score for total LE8 and its items were categorized into unfavorable score (0-49 points), intermediate score (50-79 points), and favorable score (80-100 points) according to AHA recommendation (13). For each sub-item of LE8 score: Crude model was unadjusted. Model 1 was adjusted for age, gender, and race/ethnicity. Model 2 was adjusted for age, gender, race/ethnicity, PIR, education level, alcohol consumption, marital status. In the case of total LE8 score, Model 2 was adjusted for age, gender, race/ethnicity, PIR, education level, alcohol consumption, and marital status. Results of COR (95% CI), AOR (95% CI), *P* for tend, and *P*-value presented with bold valued were statistically significant with *P*-value < 0.05 or *P*-value < 0.001.

**Figure 2:**
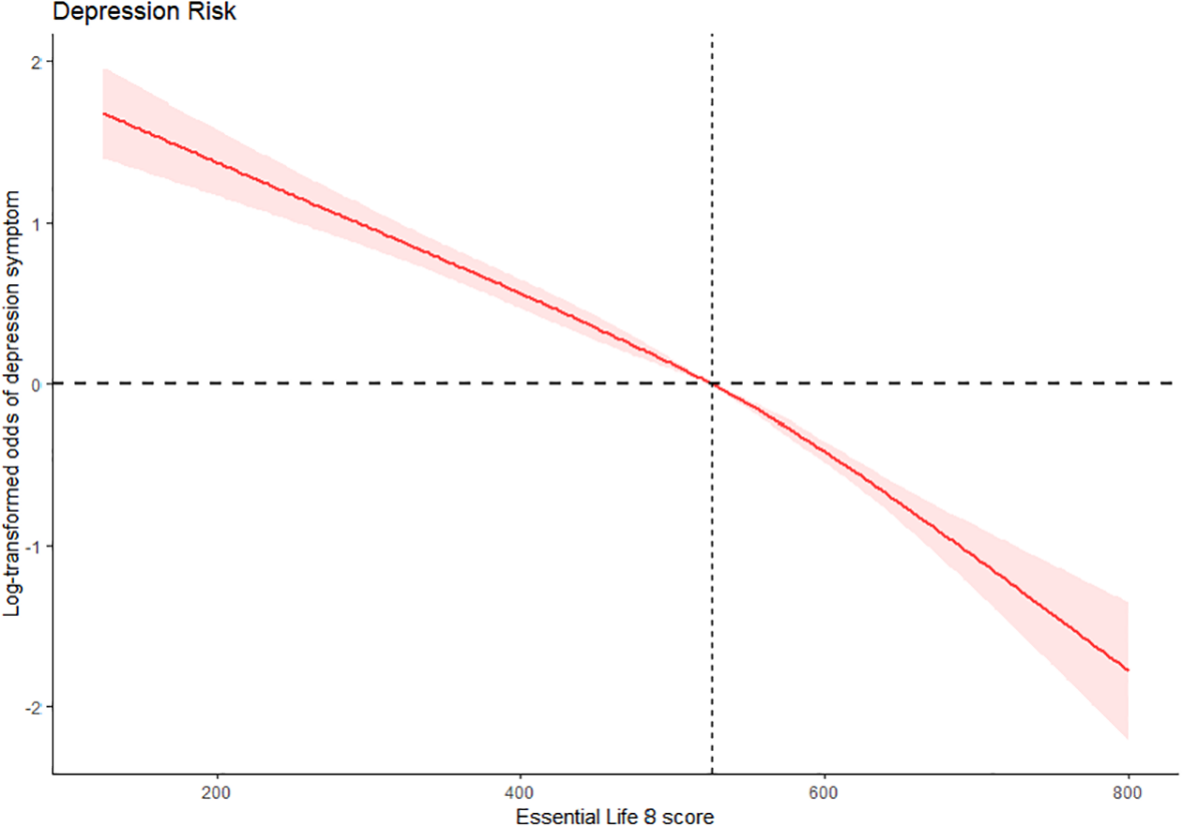
Dose-response association of total LE8 score with the risk of depression symptom, NHANES 2005-2018, U.S (N = 25,357). The method of restricted cubic splines with four knots was used to fit the dose-response relationship between total LE8 score and depressive symptom adjusting for age, gender, race/ethnicity, PIR, education level, alcohol consumption, and marital status. The red solid line and red area represent OR and 95% CI, respectively. CI, Confidence interval; LE8, Life's Essential 8; NHANES, National Health and Nutrition Examination Survey. OR, Odds ratio; PIR, Poverty-to-income ratio.

Participants with a higher total LE8 score exhibited lower odds of developing depressive symptoms when compared to those with unfavorable scores (P for trend < 0.001). Specifically, individuals with favorable scores had the lowest risk of depressive symptoms even after adjusting for all relevant factors (AOR=0.216, 95% CI: 0.178, 0.260) (**Table 2**). A dose-response relationship was found between the continuous total LE8 score and the risk of depression symptoms. The risk of depression symptoms decreased as the total LE8 score increased, as shown in **Figure 2**. Following full adjustment, a significant negative linear trend was observed in the relationship between the cumulative number of favorable LE8 components and the risk of depressive symptoms. Participants who were exposed to six or more favorable LE8 components were less likely to experience depression symptoms compared to those who did not meet any of the favorable components (*P* for trend < 0.001).

### Subgroup and sensitivity analyses

Analyses on three levels of total LE8 scores and depressive symptom stratified by demographic variables were presented in **Table 3**. Individuals aged 20-79, non-Hispanic White, current alcoholics, individuals of all educational levels, and individuals who were married or living with partners, especially those with higher total LE8 scores, are the ones who can benefit the most from LE8 (P for interaction < 0.05).

**Table 3:** Association between levels of total LE8 score and risk of depression stratified by basic demographic variables, NHANES 2005-2018, U.S (n = 25,357).

Subgroup items	Cases/participants	Total LE8 score, AOR (95% CI)			*P* for trend	*P* for interaction
		Unfavorable score (0-49 points)	Intermediate score (50-79 points)	Favorable score (80-100 points)		
Age, years						
20-39	679/8,203	Reference	0.472 (0.379,0.592)^**^	0.243 (0.181,0.327)^**^	**< 0.001**	**< 0.001**
40-59	912/8,571	Reference	0.423 (0.361,0.496)^**^	0.196 (0.140,0.269)^**^	**< 0.001**	
60-79	539/7,142	Reference	0.482(0.399,0.583)^**^	0.158 (0.080,0.282)^**^	**< 0.001**	
≥ 80	64/1,441	Reference	0.930 (0.504,1.819)	0.928 (0.251,2.780)	0.843	
Gender						
Male	807/12.431	Reference	0.468 (0.397,0.554)^**^	0.232 (0.164,0.322)^**^	**< 0.001**	0.811
Female	1,387/12,926	Reference	0.445 (0.389,0.510)^**^	0.200 (0.158,0.252)^**^	**< 0.001**	
Race/ethnicity						
Mexican American	308/3,674	Reference	0.573 (0.428,0.772)^**^	0.354 (0.219,0.562)^**^	**< 0.001**	**< 0.001**
Non-Hispanic Black	478/5,337	Reference	0.515 (0.416,0.638)^**^	0.337 (0.215,0.512)^**^	**< 0.001**	
Non-Hispanic White	985/11,681	Reference	0.413 (0.354,0.482)^**^	0.174 (0.127,0.233)^**^	**< 0.001**	
Other races	423/4,665	Reference	0.458 (0.355,0.595)^**^	0.195 (0.129,0.290)^**^	**< 0.001**	
PIR						
≥ 300%	394/9,838	Reference	0.447 (0.343,0.586)^**^	0.211 (0.143,0.308)^**^	**< 0.001**	0.602
< 300%	1,800/15,519	Reference	0.467 (0.417,0.523)^**^	0.219 (0.174,0.273)^**^	**< 0.001**	
Education level						
Less than 9th grade	272/2,165	Reference	0.517 (0.387,0.692)^**^	0.187 (0.088,0.362)^**^	**< 0.001**	**< 0.001**
9-11th grade (including 12th grade with no diploma)	459/3,384	Reference	0.553 (0.442,0.692)^**^	0.378 (0.235,0.589)^**^	**< 0.001**	
High school grade/GED or equivalent	541/5,833	Reference	0.407 (0.333,0.498)^**^	0.192 (0.121,0.294)^**^	**< 0.001**	
Some college or AA degree	669/7,733	Reference	0.442 (0.365,0.537)^**^	0.239 (0.173,0.327)^**^	**< 0.001**	
College graduate or above	253/6,242	Reference	0.385 (0.271,0.557)^**^	0.150 (0.093,0.239)^**^	**< 0.001**	
Alcohol consumption						
Never	247/3,367	Reference	0.588 (0.428,0.814)^**^	0.243 (0.138,0.414)^**^	**< 0.001**	**< 0.05**
Former	500/4,239	Reference	0.464 (0.378,0.571)^**^	0.271 (0.162,0.432)^**^	**< 0.001**	
Current	1,447/17,751	Reference	0.435 (0.381,0.496)^**^	0.205 (0.163,0.256)^**^	**< 0.001**	
Marital status						
Married/Living with partner	1,011/15,371	Reference	0.378 (0.326,0.439)^**^	0.171 (0.128,0.225)^**^	**< 0.001**	**< 0.001**
Never married	442/4,420	Reference	0.616 (0.475,0.803)^**^	0.319 (0.219,0.461)^**^	**< 0.001**	
Widowed/Divorced/Separated	741/5,566	Reference	0.519 (0.435,0.619)^**^	0.250 (0.167,0.364)^**^	**< 0.001**	

The total LE8 score was calculated as the mean of the sum of all 8 items of LE8 score and similarly ranged from 0 (if the mean score of all items was 0) to 100 (optimal CVH). The total LE8 score was categorized into unfavorable score (0-49 points), intermediate score (50-79 points), and favorable score (80-100 points) according to AHA recommendation (13). Multivariable model was adjusted for age, gender, race/ethnicity, PIR, education level, alcohol consumption, and marital status. Results of AOR (95% CI), *P* for trend, and *P* for interaction presented with bold valued were statistically significant with *P*-value < 0.05 or *P*-value < 0.001.

^**^ *P*-value < 0.001.

**Supplementary Tables S7**-**S9** presented the results of different levels of individual and total LE8 scores in relation to the risk of depression symptom, stratified by gender, race/ethnicity, and survey waves. The study indicated that there was no significant difference in the association between LE8 and depression symptoms based on gender. Furthermore, the results of the sensitivity analyses were found to be in line with the main analyses (**Supplementary Tables S10**-**S14**). Even after controlling for survey waves, hypertension, total energy intake, and antidepressant use among participants, the results remained stable and did not reach statistical significance. Utilizing the median as a threshold to categorize participants and considering the favorable score as a benchmark, binary categorical variables were created for the eight sub-terms. This method also produced consistent results, suggesting that individuals with elevated levels of both individual and total LE8 scores had a lower likelihood of experiencing symptoms of depression.

## Discussion

Based on a nationally representative sample of the U.S. population, our findings suggest that adults with higher levels of total, individual, or combined cardiovascular health metrics in LE8 were less likely to experience depression symptom. Having six or more of the eight ideal CVH components established by the AHA was linked to a lower likelihood of experiencing depressive symptom, in comparison to individuals who did not meet any of the positive components. Additionally, there was an observed 82% decrease in depression symptoms with each increase in total CVH metric score, showing a linear relationship [Six or more of the eight ideal CVH components (AOR=0.187, 95% CI: 0.133, 0.264), **Supplementary Table S13**].

Consistent with previous research, various modifiable factors of LE8 have been investigated in relation to depressive symptoms. Participation in physical activity, whether as a standalone option or in conjunction with other treatments, has been associated with a lower risk of developing depression (1, 23). PA has been demonstrated to elevate the levels of brain-derived neurotrophic factor (BDNF) (28), a neurotrophin essential for neuroplasticity, neuronal development, and viability (29). Individuals with depression often exhibit reduced levels of BDNF, which tend to rise after effective antidepressant therapy. Our previous research showed that participants who met the recommended level of physical activity and maintained a healthy diet, both key components of LE8, experienced a synergistic improvement in their symptoms of depression (24). Additionally, our study found that engaging in other aspects of LE8, such as promoting good sleep and reducing nicotine exposure, had a positive impact on reducing the risk of depression symptoms. This suggests a potential synergistic effect of a comprehensive Lifestyle Medicine (LM) approach in preventing depression, which integrates nutrition, physical activity, and other behavior management techniques (30). This hypothesis was supported by the results of a meta-analysis that examined the effects of multicomponent lifestyle modification interventions, including PA, dietary quality, and smoking cessation, on reducing symptoms of depression. The study found that individuals with depression may gain significant benefits from lifestyle modification interventions (31).

In relation to the other four biological indicators of LE8, including BMI, blood lipids, blood glucose, and blood pressure at optimal levels, our study indicates that maintaining a favorable level of BMI and blood glucose is linked to a reduced risk of depression. Moreover, an intermediate blood pressure score also shows potential benefits. Nevertheless, we could not establish whether exposure to intermediate or favorable blood lipid levels has any impact on depression. These findings are consistent with previous research (32–34), although there are conflicting results regarding cholesterol. While one longitudinal study found a negative relationship between total cholesterol (TC) and depression (9), another prospective study did not find such a correlation (12). These apparently conflicting results could be due to various factors related to differences in study designs, including variations in sample size, population groups, adjusted models, and follow-up time. Our study adds to the cumulative disadvantage theory by pointing out a notable link between discrepancies in LE8 and self-reported symptoms of depression. These findings underscore the significance of addressing lifestyle choices and advocating for health equity in order to mitigate mental health inequalities (27, 35).

Based on the analysis of gender and race/ethnicity groups, the age-adjusted prevalence of depression symptoms was higher in non-Hispanic Black participants (9.8%) compared to non-Hispanic White participants (7.4%). However, there was notable variability in the CVH score category among different race groups, particularly in the favorable group [non-Hispanic White (73.9%) vs. non-Hispanic Black (5.6%)]. Additionally, a potential race difference or significant interaction was observed in the association between LE8 and depression symptoms. A higher percentage of females (9.6%) experienced depression symptoms compared to male participants (6.2%). However, no significant difference was found in the relationship between LE8 and depression symptoms based on gender. Overall, these findings suggest that LE8 can help reduce the risk of depression across different demographic groups. Our study discovered a dose-response relationship between ideal LE8 and a decrease in the prevalence of depression symptoms, which is consistent with previous evidence showing that higher levels of cardiovascular health are associated with lower odds of developing depression symptoms. In our sensitivity analysis, we calculated the cumulative number of LE8 components for depression symptoms and found several positive associations between the cumulative number of favorable LE8s and depressive symptoms. Participants who were exposed to a higher number of favorable LE8 elements were found to have a significantly lower risk of experiencing depression symptoms, even after adjusting for covariates. The trend association indicates that any improvement in CVH score has a positive impact on depression, even for individuals with fewer than eight LE8 elements. This suggests that having some elements is better than having none. These results underscore the significance of incorporating more LE8 components simultaneously to effectively prevent depression in individuals.

## Strengthens and limitations

This study is the first to examine the changes in the prevalence of depressive symptoms from 2005 to 2018 and their association with CVH in a representative sample of adults, using the LE8 measure. Furthermore, we explored the correlation between the overall CVH score and depressive symptoms, taking into account the dose-response relationship. Stratified analyses were also conducted by gender and different racial/ethnic groups to confirm this relationship.

Several limitations should be acknowledged in this study. Firstly, the findings may not be broadly applicable to other racial groups due to the predominance of non-Hispanic White participants, representing over 46.1% of the sample. Secondly, while the 9-item version of PHQ-9 is commonly used and has demonstrated high sensitivity and specificity in initial screenings for the general population, it is essential to recognize that self-reported PHQ-9 scores may introduce measurement bias. Furthermore, potential inaccuracies in data related to diet, physical activity, nicotine exposure, and sleep health in LE8 may arise from recall and social desirability biases as data collection was based on self-reports. Thirdly, despite adjusting for significant pre-defined confounders in the multivariable model, residual confounding could still impact the results. Moreover, being an observational study, it is crucial to understand that establishing a cause-effect relationship between depressive symptoms and LE8 is challenging due to the bidirectional nature of their relationship. Finally, the absence of control for depression-related comorbidity variables in our regression model, particularly those pertaining to cardiovascular health, is noteworthy. This oversight is significant because specific cardiovascular disorders and their related comorbidities, such as heart failure, may exert an influence on physical activity, sleep duration, and depression. Therefore, future research should prospectively investigate the trajectory of LE8 components and the occurrence of depressive symptoms.

## Conclusion

Our study findings indicate a correlation between maintaining a high CVH score, as defined by the LE8 score, and a lower risk of depressive symptoms among adults in the U.S. This association was evident not only in the general population but also notably among women. Future interventional studies are necessary to confirm our primary discovery and assess whether enhancing cardiovascular health can mitigate the risk of depression onset. These studies should specifically investigate the influence of changes in depression symptoms on the LE8 score over time, employing a time-lag methodology.

## Data Availability

The original contributions presented in the study are included in the article/**Supplementary Material**. Further inquiries can be directed to the corresponding author/s.
