# Supplementary material for: Association between the American Heart Association’s New Life’s Essential 8 Metrics and Depression Symptom in U.S General Adults, Finding from NHANES 2005-2018
Source: Front Psychiatry. 2024 Nov 18;15:1480036. doi: 10.3389/fpsyt.2024.1480036 (PMC11609172; doi:10.3389/fpsyt.2024.1480036)
Supplement: Supplementary file 1 [file DataSheet1.docx]

| **Supplementary Table S1** Pairwise correlations between items of LE8 score in U.S adults aged 20 and above, NHANES 2005-2018 (n = 25,357) | | | | | | | | |
| --- | --- | --- | --- | --- | --- | --- | --- | --- |
| **Items of LE8 score** | **Diet** | **PA** | **Nicotine  exposure** | **Sleep health** | **BMI** | **Blood lipids (non–HDL-C)** | **Blood glucose** | **Blood pressure** |
| **Diet** | **1.000^**^** |  |  |  |  |  |  |  |
| **PA** | **0.060^**^** | **1.000^**^** |  |  |  |  |  |  |
| **Nicotine exposure** | **0.158^**^** | 0.010 | **1.000^**^** |  |  |  |  |  |
| **Sleep health** | **0.087^**^** | **0.036^**^** | **0.076^**^** | **1.000^**^** |  |  |  |  |
| **BMI** | **0.079^**^** | **0.078^**^** | **-0.024^**^** | **0.060^**^** | **1.000^**^** |  |  |  |
| **Blood lipids (non-HDL-C)** | **0.027^**^** | **0.035^**^** | **0.030^**^** | **0.012^*^** | **0.150^**^** | **1.000^**^** |  |  |
| **Blood glucose** | **-0.021^*^** | **0.135^**^** | **0.032^*^** | **0.048^**^** | **0.240^**^** | **0.125^**^** | **1.000^**^** |  |
| **Blood pressure** | **-0.032^**^** | **0.088^**^** | **0.028^*^** | **0.027^**^** | **0.161^**^** | **0.117^**^** | **0.241^**^** | **1.000^**^** |
| Footnotes: Correlations were estimated using Cramér's V for tricategorical variables. ^*^ *P*-value < 0.05, ^**^ *P*-value < 0.001. | | | | | | | | |
| Abbreviations: BMI, Body mass index; LE8, Life’s Essential 8; NHANES, National Health and Nutrition Examination Survey; Non-HDL-C, Non-high-density lipoprotein cholesterol; PA, Physical activity. | | | | | | | | |

| **Supplementary Table S2** Definitions of LE8 for CVH metrics and scoring algorithm | | | | | |
| --- | --- | --- | --- | --- | --- |
| **Domains** | **Items of LE8** | **Method of measurement** | **Definition** | **Quantification of CVH metric^[2]^** | |
|  |  |  |  | **Points** | **Metric range** |
| Health behaviors | Diet | Self-reported | Two interviewer-administered nonconsecutive 24-hour recalls for daily intake. | 0 | 1st - 24th percentile (bottom/least ideal quartile) |
|  |  |  |  | 25 | 25th - 49th percentile |
|  |  |  |  | 50 | 50th - 74th percentile |
|  |  |  |  | 80 | 75th - 94th percentile |
|  |  |  |  | 100 | >= 95th percentile (top/ideal diet) |
|  | PA | Self-reported | Frequency and duration of moderate or vigorous PA per week. | 0 | 0 minutes |
|  |  |  |  | 20 | 1 - 29 minutes |
|  |  |  |  | 40 | 30 - 59 minutes |
|  |  |  |  | 60 | 60 - 89 minutes |
|  |  |  |  | 80 | 90 - 119 minutes |
|  |  |  |  | 90 | 120 - 149 minutes |
|  |  |  |  | 100 | >= 150 minutes |
|  | Nicotine exposure | Self-reported | Use of ombustible tobacco or inhalation nicotine delivery systems. Subtract 20 points (unless score is 0) for living with active indoor smoker in home. | 0 | Current smoker |
|  |  |  |  | 25 | Former smoker, quit < 1 y |
|  |  |  |  | 50 | Former smoker, quit 1 - <5 y |
|  |  |  |  | 75 | Former smoker, quit >= 5 y |
|  |  |  |  | 100 | Never smoker |
|  | Sleep health | Self-reported | Average hours of sleep per night. | 0 | < 4 hours |
|  |  |  |  | 20 | 4 - < 5 hours |
|  |  |  |  | 40 | 5 - < 6 or >= 10 hours |
|  |  |  |  | 70 | 6 - < 7 hours |
|  |  |  |  | 90 | 9 - < 10 hours |
|  |  |  |  | 100 | 7 - < 9 hours |
| Biological indicators | BMI | Examination based | Body weight (kilograms) divided by height squared (meters squared). | 0 | >= 40.0 kg/m^2^ |
|  |  |  |  | 15 | 35.0 - 39.9 kg/m^2^ |
|  |  |  |  | 30 | 30.0 - 34.9 kg/m^2^ |
|  |  |  |  | 70 | 25.5 - 29.9 kg/m^2^ |
|  |  |  |  | 100 | < 25 kg/m^2^ |
|  | Blood lipids (non-HDL-C) | Examination based | Subtract HDL-C from TC to get non-HDL-C (mg/dL). If drug-treated level, subtract 20 points. | 0 | >= 220 mg/dL |
|  |  |  |  | 20 | 190 - 219 mg/dL |
|  |  |  |  | 40 | 160 - 189 mg/dL |
|  |  |  |  | 60 | 130 - 159 mg/dL |
|  |  |  |  | 100 | < 130 mg/dL |
|  | Blood glucose | Examination based | FBG was measured based on fasting blood samples. HbA1c levels were determined based on both fasting and non-fasting blood samples. | 0 | Diabetes with HbA1c >= 10.0 % |
|  |  |  |  | 10 | Diabetes with HbA1c 9.0 - 9.9 % |
|  |  |  |  | 20 | Diabetes with HbA1c 8.0 - 8.9 % |
|  |  |  |  | 30 | Diabetes with HbA1c 7.0 - 7.9 % |
|  |  |  |  | 40 | Diabetes with HbA1c < 7.0 % |
|  |  |  |  | 60 | No history of diabetes and HbA1c 5.7 - 6.4 (prediabetes) |
|  |  |  |  | 100 | No history of diabetes and HbA1c < 5.7 % |
|  | Blood pressure | Examination based | SBP and DBP were measured twice by an Omron device, and averaged values were used. Subtract 20 points if treated level. | 0 | >= 160 mm Hg or >= 100 mm Hg |
|  |  |  |  | 25 | 140 - 159 mm Hg or 90 - 99 mm Hg |
|  |  |  |  | 50 | 130 - 139 mm Hg or 80 - 89 mm Hg (stage 1 hypertension) |
|  |  |  |  | 75 | 120 - 129 mm Hg / < 80mm Hg |
|  |  |  |  | 100 | < 120 mm Hg / < 80 mm Hg (Optimal) |
| Footnotes: The LE8, introduced by AHA, is a quantitative metric of CVH, that comprises two domains: health behaviors (diet, PA, nicotine exposure, and sleep health), and biological indicators (BMI, blood lipids, blood glucose, and blood pressure)^[1]^. Diet quality was assessed based on the Healthy Eating Index 2015, the mean values of each dietary component collected from 2 interviewer-administered nonconsecutive 24-hour dietary recalls were used for the assessment. Frequency and duration of moderate or vigorous PA, nicotine exposure, and sleep duration were assessed by self-reported questionnaires at baseline. Height and weight were also measured at initial review, and BMI (kg/m^2^) was calculated as weight (kilograms) divided by height squared (meters). TC was measured by the CHO-POD analysis, and HDL-C was measured by enzyme immunoinhibition on a Backman Coulter AU5800. The concentration of non-HDL-C was calculated by subtracting HDL-C from TC. FBG was measured based on fasting blood samples. HbA1c levels were determined using HPLC analysis on a Bio-Rad VARIANT II Turbo, which involved analyzing both fasting and non-fasting blood samples. SBP and DBP were measured twice by an Omron device, and average values were used. ^[1]^ Lloyd-Jones DM, Allen NB, Anderson CAM, et al. Life's Essential 8: Updating and Enhancing the American Heart Association's Construct of Cardiovascular Health: A Presidential Advisory From the American Heart Association. Circulation. 2022;146(5): e18-e43. doi:10.1161/CIR.0000000000001078 ^[2]^ Li X, Ma H, Wang X, Feng H, Qi L. Life's Essential 8, Genetic Susceptibility, and Incident Cardiovascular Disease: A Prospective Study. Arterioscler Thromb Vasc Biol. 2023;43(7):1324-1333. doi:10.1161/ATVBAHA.123.319290 | | | | | |
| Abbreviations: AHA, American Heart Association; BMI, Body mass index; CHO-POD, Cholesterol oxidase-perox-idase; CVH, Cardiovascular health; DBP, Diastolic blood pressure; FBG, Fasting blood glucose; HbA1c, Glycated hemoglobin; HDL-C, High-density lipoprotein cholesterol; HPLC, High performance liquid chromatography; LE8, Life’s Essential 8; Non-HDL-C, Non-high-density lipoprotein cholesterol; PA, Physical activity; SBP, Systolic blood pressure; TC, Total cholesterol. | | | | | |

| **Supplementary Table S3** Survey-weighted characteristic variables of the study participants stratified by gender, NHANES 2005-2018, U.S (n = 25,357) | | | | | | |
| --- | --- | --- | --- | --- | --- | --- |
| **Characteristic variables** | **Age-adjusted prevalence rate^a^** | **Estimate U.S population (n)** | **Total participants**  **[n (%)]** | **Gender** | | ***P*-value^b^** |
|  |  |  |  | **Male** | **Female** |  |
| **No. of participants** | 7.9 (0.3) | 1,184,141,254 | 25,357 (100.0) | 12,431 (49.0) | 12,926 (51.0) | - |
| **Age, years** | - | - | 47.98 ± 0.26 | 47.09 ± 0.29 | 48.81 ± 0.28 | **< 0.001** |
| 20-39 | 7.9 (0.4) | 408,025,337 | 8,203 (32.4) | 4,114 (36.8) | 4,089 (32.3) | **< 0.001** |
| 40-59 | 9.3 (0.5) | 459,397,489 | 8,571 (33.8) | 4,061 (38.3) | 4,510 (39.3) |  |
| 60-79 | 6.2 (0.5) | 271,522,095 | 7,142 (28.2) | 3,537 (21.7) | 3,605 (24.1) |  |
| ≥ 80 | 4.1 (0.6) | 45,196,334 | 1,441 (5.7) | 719 (3.2) | 722 (4.36) |  |
| **Race/ethnicity** |  |  |  |  |  |  |
| Mexican American | 8.6 (0.7) | 89,101,932 | 3,674 (14.5) | 1,798 (8.1) | 1,876 (7.0) | **< 0.001** |
| Non-Hispanic Black | 9.8 (0.5) | 124,176,625 | 5,337 (21.1) | 2,557 (9.8) | 2,780 (11.1) |  |
| Non-Hispanic White | 7.4 (0.4) | 833,102,497 | 11,681 (46.1) | 5,834 (70.3) | 5,847 (70.4) |  |
| Other races | 9.8 (0.8) | 137,760,201 | 4,665 (18.4) | 2,242 (11.8) | 2,423 (11.5) |  |
| **BMI, kg/m^2^** | - | - | 29.12 ± 0.09 | 28.97 ± 0.10 | 29.25 ± 0.13 | **< 0.05** |
| **PIR** | - | - | 3.07 ± 0.04 | 3.15 ± 0.04 | 2.99 ± 0.04 | **< 0.001** |
| ≥ 300% | 4.2 (0.3) | 611,632,294 | 9,838 (38.8) | 5,026 (53.6) | 4,812 (49.8) | **< 0.001** |
| < 300% | 12.4 (0.5) | 572,508,960 | 15,519 (61.2) | 7,405 (46.4) | 8,114 (50.2) |  |
| **Education level** |  |  |  |  |  |  |
| Less than 9th grade | 13.2 (1.3) | 48,957,898 | 2,165 (8.5) | 1,148 (4.5) | 1,017 (3.8) | **< 0.05** |
| 9-11th grade (including 12th grade with no diploma) | 13.4 (0.9) | 114,617,982 | 3,384 (13.4) | 1,713 (9.8) | 1,671 (9.6) |  |
| High school grade/GED or equivalent | 9.7 (0.6) | 275,421,825 | 5,833 (23.0) | 2,977 (23.9) | 2,856 (22.6) |  |
| Some college or AA degree | 8.6 (0.5) | 380,180,823 | 7,733 (30.5) | 3,501 (30.9) | 4,232 (33.3) |  |
| College graduate or above | 3.6 (0.4) | 364,962,726 | 6,242 (24.6) | 3,092 (31.0) | 3,150 (30.7) |  |
| **Alcohol consumption** |  |  |  |  |  |  |
| Never | 6.5 (0.6) | 122,416,118 | 3,367 (13.3) | 925 (6.5) | 2,442 (14.0) | **< 0.001** |
| Former | 11.1 (0.9) | 160,448,474 | 4,239 (16.7) | 2,154 (13.5) | 2,085 (13.6) |  |
| Current | 7.5 (0.3) | 901,276,662 | 17,751 (70.0) | 9,352 (80.1) | 8,399 (72.5) |  |
| **Marital status** |  |  |  |  |  |  |
| Married/Living with partner | 5.9 (0.3) | 752,598,329 | 15,371 (60.6) | 8,315 (67.1) | 7,056 (60.3) | **< 0.001** |
| Never married | 10.4 (0.8) | 209,613,147 | 4,420 (17.4) | 2,202 (20.0) | 2,218 (15.6) |  |
| Widowed/Divorced/Separated | 14.9 (0.9) | 221,929,779 | 5,566 (22.0) | 1,914 (12.9) | 3,652 (24.2) |  |
| **Items of LE8 score (0-100)** |  |  |  |  |  |  |
| **Diet** | - | - | 39.44 ± 0.51 | 36.26 ± 0.54 | 42.40 ± 0.61 | **< 0.001** |
| Unfavorable score | 10.1 (0.5) | 599,051,916 | 12,782 (50.4) | 6,728 (55.1) | 6,054 (46.4) | **< 0.001** |
| Intermediate score | 6.3 (0.4) | 289,423,345 | 6,302 (24.9) | 3,024 (23.3) | 3,278 (25.5) |  |
| Favorable score | 5.0 (0.4) | 295,665,993 | 6,273 (24.7) | 2,679 (21.6) | 3,594 (28.1) |  |
| **PA** | - | - | 71.54 ± 0.50 | 76.52 ± 0.57 | 66.90 ± 0.64 | **< 0.001** |
| Unfavorable score | 12.3 (0.6) | 324,089,678 | 8,083 (31.9) | 3,383 (22.8) | 4,700 (31.7) | **< 0.001** |
| Intermediate score | 7.2 (1.2) | 59,532,580 | 1,206 (4.8) | 521 (4.4) | 685 (5.7) |  |
| Favorable score | 6.3 (0.3) | 800,518,996 | 16,068 (63.4) | 8,527 (72.9) | 7,541 (62.7) |  |
| **Nicotine exposure** | - | - | 71.10 ± 0.49 | 67.59 ± 0.63 | 74.38 ± 0.58 | **< 0.001** |
| Unfavorable score | 14.7 (0.7) | 265,696,864 | 5,779 (22.8) | 3,310 (25.1) | 2,469 (20.0) | **< 0.001** |
| Intermediate score | 6.5 (0.6) | 268,062,407 | 5,707 (22.5) | 3,411 (26.3) | 2,296 (19.3) |  |
| Favorable score | 5.4 (0.3) | 650,381,983 | 13,871 (54.7) | 5,710 (48.7) | 8,161 (60.8) |  |
| **Sleep health** | - | - | 83.35 ± 0.29 | 83.15 ± 0.34 | 83.54 ± 0.36 | 0.320 |
| Unfavorable score | 19.0 (0.9) | 175,478,530 | 4,467 (17.6) | 2,176 (14.4) | 2,291 (15.2) | **< 0.001** |
| Intermediate score | 7.5 (0.6) | 241,448,635 | 5,456 (21.5) | 2,828 (22.6) | 2,628 (18.4) |  |
| Favorable score | 5.6 (0.3) | 767,214,089 | 15,434 (60.9) | 7,427 (63.0) | 8,007 (66.4) |  |
| **BMI** | - | - | 60.08 ± 0.45 | 60.09 ± 0.54 | 60.06 ± 0.58 | 0.970 |
| Unfavorable score | 10.1 (0.4) | 448,070,779 | 9,935 (39.2) | 4,450 (36.8) | 5,485 (38.8) | **< 0.001** |
| Intermediate score | 6.5 (0.5) | 388,661,523 | 8,341 (32.9) | 4,720 (38.0) | 3,621 (28.0) |  |
| Favorable score | 6.7 (0.5) | 347,408,952 | 7,081 (27.9) | 3,261 (25.2) | 3,820 (33.2) |  |
| **Blood lipids (non–HDL-C)** | - | - | 63.49 ± 0.35 | 61.50 ± 0.44 | 65.34 ± 0.45 | **< 0.001** |
| Unfavorable score | 8.8 (0.4) | 440,283,865 | 9,330 (36.8) | 4,783 (39.6) | 4,547 (34.9) | **< 0.001** |
| Intermediate score | 6.9 (0.4) | 270,081,136 | 5,613 (22.1) | 2,730 (22.3) | 2,883 (23.3) |  |
| Favorable score | 7.7 (0.4) | 473,776,253 | 10,414 (41.1) | 4,918 (38.2) | 5,496 (41.8) |  |
| **Blood glucose** | - | - | 86.05 ± 0.25 | 85.56 ± 0.32 | 86.51 ± 0.33 | **< 0.05** |
| Unfavorable score | 11.7 (0.8) | 166,598,615 | 4,671 (18.4) | 2,387 (14.7) | 2,284 (13.5) | **< 0.001** |
| Intermediate score | 8.9 (0.8) | 201,806,038 | 5,019 (19.8) | 2,547 (17.2) | 2,472 (16.9) |  |
| Favorable score | 7.0 (0.4) | 815,736,601 | 15,667 (61.8) | 7,497 (68.1) | 8,170 (69.6) |  |
| **Blood pressure** | - | - | 69.03 ± 0.33 | 66.60 ± 0.45 | 71.30 ± 0.37 | **< 0.001** |
| Unfavorable score | 9.1 (0.7) | 249,775,713 | 6,307 (24.9) | 3,101 (21.0) | 3,206 (21.2) | **< 0.001** |
| Intermediate score | 7.3 (0.5) | 387,760,307 | 8,143 (32.1) | 4,705 (39.1) | 3,438 (26.9) |  |
| Favorable score | 7.8 (0.4) | 546,605,234 | 10,907 (43.0) | 4,625 (39.9) | 6,282 (52.0) |  |
| **Survey waves** |  |  |  |  |  |  |
| 2005-2006 | 5.9 (0.7) | 170,066,111 | 3,388 (13.4) | 1,723 (14.4) | 1,665 (14.4) | 0.630 |
| 2007-2008 | 8.6 (0.9) | 160,988,009 | 3,964 (15.6) | 1,941 (13.1) | 2,023 (14.0) |  |
| 2009-2010 | 8.0 (0.6) | 165,180,398 | 4,150 (16.4) | 2,030 (14.2) | 2,120 (13.8) |  |
| 2011-2012 | 8.2 (0.9) | 176,400,748 | 3,625 (14.3) | 1,814 (15.2) | 1,811 (14.7) |  |
| 2013-2014 | 9.1 (0.7) | 184,101,388 | 3,916 (15.4) | 1,873 (15.6) | 2,043 (15.5) |  |
| 2015-2016 | 7.8 (0.8) | 174,615,296 | 3,541 (14.0) | 1,720 (14.8) | 1,821 (14.7) |  |
| 2017-2018 | 7.7 (0.7) | 152,789,305 | 2,773 (10.9) | 1,330 (12.8) | 1,443 (13.0) |  |
| **Depressive symptom** |  |  |  |  |  |  |
| No | - | 1,090,357,765 | 23,163 (91.4) | 11,624 (93.8) | 11,539 (90.5) | **< 0.001** |
| Yes | - | 93,783,490 | 2,194 (8.7) | 807 (6.2) | 1,387 (9.5) |  |
| Footnotes: Continuous variables are presented as weighted mean ± SE, and categorical variables are presented as counting (n) and survey-weighted percentage (%). Score for items of LE8 was categorized into unfavorable score (0-49 points), intermediate score (50-79 points), and favorable score (80-100 points) according to AHA recommendation^[1]^. ^a^ Age-adjusted prevalence rates are present as [weighted number, % (SE)]. There were two steps to calculate them: First, the standard age proportions for age groups, based on the 2000 U.S Census Standard Population data, were calculated by dividing the age-specific Census population (P) by the total Census population number (T), and the standardizing proportions (P/T) should sum to 1. Second, the age-specific prevalence from the study population is multiplied by the proportion of people in that age group in the standard population, and results summed up to get the age-adjusted estimates. More detail can be got from: https://wwwn.cdc.gov/nchs/nhanes/tutorials/samplecode.aspx. ^b^ The *P*-values were assessed by *t*-test or Mann-Whitney U test (continuous variables) or by chi-square test or Fisher exact test (categorical variables) to represent the differences between male and female. *P*-values presented with bold valued were statistically significant. ^[1]^ Lloyd-Jones DM, Allen NB, Anderson CAM, et al. Life's Essential 8: Updating and Enhancing the American Heart Association's Construct of Cardiovascular Health: A Presidential Advisory From the American Heart Association. Circulation. 2022;146(5): e18-e43. doi:10.1161/CIR.0000000000001078 | | | | | | |
| Abbreviations: AA, Associate's degree; BMI, Body mass index; GED, General equivalent diploma; LE8, Life’s Essential 8; NHANES, National Health and Nutrition Examination Survey; Non-HDL-C, Non-high-density lipoprotein cholesterol; PA, Physical activity; PIR, Poverty-to-income ratio; SE, Standard error. | | | | | | |

| **Supplementary Table S4** Survey-weighted characteristic variables of the study participants stratified by race/ethnicity, NHANES 2005-2018, U.S (n = 25,357) | | | | | | | | |
| --- | --- | --- | --- | --- | --- | --- | --- | --- |
| **Characteristic variables** | **Age-adjusted prevalence rate^a^** | **Estimate U.S population (n)** | **Total participants [n (%)]** | **Race/ethnicity** | | | | ***P*-value^b^** |
|  |  |  |  | **Mexican American** | **Non-Hispanic Black** | **Non-Hispanic White** | **Other races** |  |
| **No. of participants** | 7.9 (0.3) | 1,184,141,254 | 25,357 (100.0) | 3,674 (14.5) | 5,337 (21.1) | 11,681 (46.1) | 4,665 (18.4) | - |
| **Age, years** | - | - | 47.98 ± 0.26 | 40.86 ± 0.44 | 45.00 ± 0.39 | 49.98 ± 0.30 | 43.18 ± 0.36 | **< 0.05** |
| 20-39 | 7.9 (0.4) | 408,025,337 | 8,203 (32.4) | 1,404 (52.3) | 1,678 (39.8) | 3,330 (29.8) | 1,791 (46.1) | **< 0.001** |
| 40-59 | 9.3 (0.5) | 459,397,489 | 8,571 (33.8) | 1,276 (34.3) | 1,914 (40.2) | 3,743 (39.3) | 1,638 (37.6) |  |
| 60-79 | 6.2 (0.5) | 271,522,095 | 7,142 (28.2) | 941 (12.5) | 1,611 (18.3) | 3,440 (26.0) | 1,150 (15.1) |  |
| ≥ 80 | 4.1 (0.6) | 45,196,334 | 1,441 (5.7) | 53 (0.9) | 134 (1.7) | 1,168 (4.9) | 86 (1.2) |  |
| **Gender** |  |  |  |  |  |  |  |  |
| Male | 6.2 (0.4) | 571,113,588 | 12,431 (49.0) | 1,798 (51.8) | 2,557 (45.1) | 5,834 (48.2) | 2,242 (49.0) | **< 0.001** |
| Female | 9.6 (0.4) | 613,027,666 | 12,926 (51.0) | 1,876 (48.2) | 2,780 (54.9) | 5,847 (51.8) | 2,423 (51.0) |  |
| **BMI, kg/m^2^** | - | - | 29.12 ± 0.09 | 30.26 ± 0.18 | 31.07 ± 0.16 | 28.87 ± 0.11 | 28.16 ± 0.17 | **< 0.001** |
| **PIR** | - | - | 3.07 ± 0.04 | 2.02 ± 0.05 | 2.34 ± 0.05 | 3.35 ± 0.04 | 2.71 ± 0.06 | **< 0.001** |
| ≥ 300% | 4.2 (0.3) | 611,632,294 | 9,838 (38.8) | 794 (24.2) | 1,830 (33.0) | 5,356 (58.8) | 1,858 (42.8) | **< 0.001** |
| < 300% | 12.4 (0.5) | 572,508,960 | 15,519 (61.2) | 2,880 (75.8) | 3,507 (67.0) | 6,325 (41.2) | 2,807 (57.2) |  |
| **Education level** |  |  |  |  |  |  |  |  |
| Less than 9th grade | 13.2 (1.3) | 48,957,898 | 2,165 (8.5) | 1,061 (21.7) | 207 (3.0) | 432 (2.0) | 465 (6.9) | **< 0.001** |
| 9-11th grade (including 12th grade with no diploma) | 13.4 (0.9) | 114,617,982 | 3,384 (13.4) | 725 (19.8) | 922 (16.6) | 1,238 (7.6) | 499 (9.6) |  |
| High school grade/GED or equivalent | 9.7 (0.6) | 275,421,825 | 5,833 (23.0) | 756 (22.5) | 1,366 (26.4) | 2,915 (23.6) | 796 (18.7) |  |
| Some college or AA degree | 8.6 (0.5) | 380,180,823 | 7,733 (30.5) | 804 (25.7) | 1,848 (35.5) | 3,785 (32.5) | 1,296 (30.9) |  |
| College graduate or above | 3.6 (0.4) | 364,962,726 | 6,242 (24.6) | 328 (10.4) | 994 (18.5) | 3,311 (34.3) | 1,609 (34.0) |  |
| **Alcohol consumption** |  |  |  |  |  |  |  |  |
| Never | 6.5 (0.6) | 122,416,118 | 3,367 (13.3) | 596 (14.2) | 763 (14.3) | 1,136 (8.5) | 872 (15.6) | **< 0.001** |
| Former | 11.1 (0.9) | 160,448,474 | 4,239 (16.7) | 618 (13.3) | 990 (15.1) | 2,021 (13.5) | 610 (12.5) |  |
| Current | 7.5 (0.3) | 901,276,662 | 17,751 (70.0) | 2,460 (72.6) | 3,584 (70.6) | 8,524 (78.0) | 3,183 (71.9) |  |
| **Marital status** |  |  |  |  |  |  |  |  |
| Married/Living with partner | 5.9 (0.3) | 752,598,329 | 15,371 (60.6) | 2,520 (67.5) | 2,426 (43.3) | 7,479 (66.5) | 2,946 (61.5) | **< 0.001** |
| Never married | 10.4 (0.8) | 209,613,147 | 4,420 (17.4) | 499 (18.3) | 1,470 (32.6) | 1,551 (14.6) | 900 (23.0) |  |
| Widowed/Divorced/Separated | 14.9 (0.9) | 221,929,779 | 5,566 (22.0) | 655 (14.2) | 1,441 (24.1) | 2,651 (19.0) | 819 (15.5) |  |
| **Items of LE8 score (0-100)** |  |  |  |  |  |  |  |  |
| **Diet** | - | - | 39.44 ± 0.51 | 37.14 ± 0.88 | 33.27 ± 0.78 | 39.91 ± 0.62 | 43.62 ± 0.73 | **< 0.001** |
| Unfavorable score | 10.1 (0.5) | 599,051,916 | 12,782 (50.4) | 1,805 (52.7) | 2,998 (59.3) | 6,021 (49.9) | 1,958 (45.8) | **< 0.001** |
| Intermediate score | 6.3 (0.4) | 289,423,345 | 6,302 (24.9) | 1,036 (26.7) | 1,277 (23.0) | 2,799 (24.4) | 1,190 (24.7) |  |
| Favorable score | 5.0 (0.4) | 295,665,993 | 6,273 (24.7) | 833 (20.7) | 1,062 (17.8) | 2,861 (25.8) | 1,517 (29.5) |  |
| **PA** | - | - | 71.54 ± 0.50 | 68.54 ± 1.27 | 65.83 ± 0.99 | 72.79 ± 0.60 | 71.13 ± 1.03 | **< 0.001** |
| Unfavorable score | 12.3 (0.6) | 324,089,678 | 8,083 (31.9) | 1,333 (30.5) | 1,887 (33.2) | 3,501 (26.1) | 1,362 (27.9) | **< 0.001** |
| Intermediate score | 7.2 (1.2) | 59,532,580 | 1,206 (4.8) | 167 (4.6) | 251 (4.7) | 587 (5.2) | 201 (4.3) |  |
| Favorable score | 6.3 (0.3) | 800,518,996 | 16,068 (63.4) | 2,174 (64.9) | 3,199 (62.1) | 7,593 (68.7) | 3,102 (67.8) |  |
| **Nicotine exposure** | - | - | 71.10 ± 0.49 | 76.39 ± 0.99 | 67.71 ± 0.92 | 70.38 ± 0.68 | 75.14 ± 0.93 | **< 0.001** |
| Unfavorable score | 14.7 (0.7) | 265,696,864 | 5,779 (22.8) | 633 (18.3) | 1,400 (27.4) | 2,930 (22.6) | 816 (19.9) | **< 0.001** |
| Intermediate score | 6.5 (0.6) | 268,062,407 | 5,707 (22.5) | 735 (17.1) | 921 (13.8) | 3,196 (25.5) | 855 (16.8) |  |
| Favorable score | 5.4 (0.3) | 650,381,983 | 13,871 (54.7) | 2,306 (64.6) | 3,016 (58.9) | 5,555 (51.9) | 2,994 (63.4) |  |
| **Sleep health** | - | - | 83.35 ± 0.29 | 83.29 ± 0.55 | 73.73 ± 0.53 | 84.99 ± 0.35 | 82.12 ± 0.55 | **< 0.001** |
| Unfavorable score | 19.0 (0.9) | 175,478,530 | 4,467 (17.6) | 556 (15.0) | 1,405 (26.2) | 1,740 (12.9) | 766 (16.0) | **< 0.001** |
| Intermediate score | 7.5 (0.6) | 241,448,635 | 5,456 (21.5) | 774 (21.0) | 1,371 (25.8) | 2,282 (19.3) | 1,029 (21.8) |  |
| Favorable score | 5.6 (0.3) | 767,214,089 | 15,434 (60.9) | 2,344 (64.1) | 2,561 (47.9) | 7,659 (67.8) | 2,870 (62.2) |  |
| **BMI** | - | - | 60.08 ± 0.45 | 53.59 ± 0.92 | 51.69 ± 0.68 | 61.23 ± 0.52 | 64.89 ± 0.90 | **< 0.001** |
| Unfavorable score | 10.1 (0.4) | 448,070,779 | 9,935 (39.2) | 1,610 (44.5) | 2,572 (48.8) | 4,314 (36.4) | 1,439 (32.5) | **< 0.001** |
| Intermediate score | 6.5 (0.5) | 388,661,523 | 8,341 (32.9) | 1,396 (36.5) | 1,519 (27.6) | 3,882 (33.3) | 1,544 (32.1) |  |
| Favorable score | 6.7 (0.5) | 347,408,952 | 7,081 (27.9) | 668 (19.0) | 1,246 (23.7) | 3,485 (30.3) | 1,682 (35.4) |  |
| **Blood lipids (non–HDL-C)** | - | - | 63.49 ± 0.35 | 63.11 ± 0.81 | 69.70 ± 0.55 | 62.51 ± 0.44 | 64.25 ± 0.77 | **< 0.05** |
| Unfavorable score | 8.8 (0.4) | 440,283,865 | 9,330 (36.8) | 1,425 (36.7) | 1,768 (32.4) | 4,384 (38.0) | 1,753 (36.8) | **< 0.001** |
| Intermediate score | 6.9 (0.4) | 270,081,136 | 5,613 (22.1) | 920 (25.9) | 1,086 (21.4) | 2,532 (22.7) | 1,075 (22.8) |  |
| Favorable score | 7.7 (0.4) | 473,776,253 | 10,414 (41.1) | 1,329 (37.5) | 2,483 (46.2) | 4,765 (39.3) | 1,837 (40.4) |  |
| **Blood glucose** | - | - | 86.05 ± 0.25 | 84.62 ± 0.74 | 80.00 ± 0.53 | 87.30 ± 0.31 | 84.58 ± 0.50 | 0.230 |
| Unfavorable score | 11.7 (0.8) | 166,598,615 | 4,671 (18.4) | 769 (15.5) | 1,324 (21.6) | 1,726 (12.5) | 852 (16.0) | **< 0.001** |
| Intermediate score | 8.9 (0.8) | 201,806,038 | 5,019 (19.8) | 711 (17.4) | 1,260 (22.7) | 2,100 (15.9) | 948 (18.5) |  |
| Favorable score | 7.0 (0.4) | 815,736,601 | 15,667 (61.8) | 2,194 (67.1) | 2,753 (55.7) | 7,855 (71.6) | 2,865 (65.5) |  |
| **Blood pressure** | - | - | 69.03 ± 0.33 | 74.86 ± 0.71 | 63.30 ± 0.61 | 68.52 ± 0.42 | 73.48 ± 0.65 | **< 0.001** |
| Unfavorable score | 9.1 (0.7) | 249,775,713 | 6,307 (24.9) | 698 (15.1) | 1,744 (28.0) | 2,902 (21.4) | 963 (16.8) | 0.070 |
| Intermediate score | 7.3 (0.5) | 387,760,307 | 8,143 (32.1) | 1,241 (33.0) | 1,720 (33.1) | 3,708 (32.8) | 1,474 (32.2) |  |
| Favorable score | 7.8 (0.4) | 546,605,234 | 10,907 (43.0) | 1,735 (51.9) | 1,873 (38.8) | 5,071 (45.8) | 2,228 (51.0) |  |
| **Survey waves** |  |  |  |  |  |  |  |  |
| 2005-2006 | 5.9 (0.7) | 170,066,111 | 3,388 (13.4) | 617 (12.9) | 747 (14.2) | 1,796 (15.4) | 228 (9.2) | 0.120 |
| 2007-2008 | 8.6 (0.9) | 160,988,009 | 3,964 (15.6) | 625 (13.4) | 792 (13.6) | 2,020 (14.2) | 527 (10.2) |  |
| 2009-2010 | 8.0 (0.6) | 165,180,398 | 4,150 (16.4) | 698 (13.9) | 701 (13.4) | 2,188 (14.4) | 563 (11.8) |  |
| 2011-2012 | 8.2 (0.9) | 176,400,748 | 3,625 (14.3) | 331 (13.8) | 938 (15.3) | 1,486 (14.7) | 870 (16.3) |  |
| 2013-2014 | 9.1 (0.7) | 184,101,388 | 3,916 (15.4) | 483 (17.0) | 776 (16.0) | 1,823 (15.1) | 834 (17.1) |  |
| 2015-2016 | 7.8 (0.8) | 174,615,296 | 3,541 (14.0) | 579 (15.3) | 730 (14.0) | 1,341 (14.4) | 891 (17.4) |  |
| 2017-2018 | 7.7 (0.7) | 152,789,305 | 2,773 (10.9) | 341 (13.6) | 653 (13.4) | 1,027 (11.9) | 752 (18.1) |  |
| **Depressive symptom** |  |  |  |  |  |  |  |  |
| No | - | 1,090,357,765 | 23,163 (91.4) | 3,366 (91.9) | 4,859 (90.0) | 10,696 (92.7) | 4,242 (90.2) | **< 0.001** |
| Yes | - | 93,783,490 | 2,194 (8.7) | 308 (8.1) | 478 (10.0) | 985 (7.3) | 423 (9.9) |  |
| Footnotes: Continuous variables are presented as weighted mean ± SE, and categorical variables are presented as counting (n) and survey-weighted percentage (%). Score for items of LE8 was categorized into unfavorable score (0-49 points), intermediate score (50-79 points), and favorable score (80-100 points) according to AHA recommendation^[1]^. ^a^ Age-adjusted prevalence rates are present as [weighted number, % (SE)]. There were two steps to calculate them: First, the standard age proportions for age groups, based on the 2000 U.S Census Standard Population data, were calculated by dividing the age-specific Census population (P) by the total Census population number (T), and the standardizing proportions (P/T) should sum to 1. Second, the age-specific prevalence from the study population is multiplied by the proportion of people in that age group in the standard population, and results summed up to get the age-adjusted estimates. More detail can be got from: https://wwwn.cdc.gov/nchs/nhanes/tutorials/samplecode.aspx. ^b^ The *P*-values were assessed by one-way ANOVA (continuous variables) or by chi-square test (categorical variables) to represent the differences among four groups of race/ethnicity. *P*-values presented with bold valued were statistically significant. ^[1]^ Lloyd-Jones DM, Allen NB, Anderson CAM, et al. Life's Essential 8: Updating and Enhancing the American Heart Association's Construct of Cardiovascular Health: A Presidential Advisory From the American Heart Association. Circulation. 2022;146(5): e18-e43. doi:10.1161/CIR.0000000000001078 | | | | | | | | |
| Abbreviations: AA, Associate's degree; ANOVA, Analysis of variance; BMI, Body mass index; GED, General equivalent diploma; LE8, Life’s Essential 8; NHANES, National Health and Nutrition Examination Survey; Non-HDL-C, Non-high-density lipoprotein cholesterol; PA, Physical activity; PIR, Poverty-to-income ratio; SE, Standard error. | | | | | | | | |

| **Supplementary Table S5** Survey-weighted characteristic variables of the study participants stratified by depressive symptom, NHANES 2005-2018, U.S (n = 25,357) | | | | | | |
| --- | --- | --- | --- | --- | --- | --- |
| **Characteristic variables** | **Age-adjusted prevalence rate^a^** | **Estimate U.S population (n)** | **Total participants**  **[n (%)]** | **Depressive symptom** | | ***P*-value^b^** |
|  |  |  |  | **No** | **Yes** |  |
| **No. of participants** | 7.9 (0.3) | 1,184,141,254 | 25,357 (100.0) | 23,163 (91.4) | 2,194 (8.7) | - |
| **Age, years** | - | - | 47.98 ± 0.26 | 48.10 ± 0.27 | 46.59 ± 0.47 | **< 0.05** |
| 20-39 | 7.9 (0.4) | 408,025,337 | 8,203 (32.4) | 7,524 (34.5) | 679 (34.5) | **< 0.001** |
| 40-59 | 9.3 (0.5) | 459,397,489 | 8,571 (33.8) | 7,659 (38.2) | 912 (45.5) |  |
| 60-79 | 6.2 (0.5) | 271,522,095 | 7,142 (28.2) | 6,603 (23.4) | 539 (18.0) |  |
| ≥ 80 | 4.1 (0.6) | 45,196,334 | 1,441 (5.7) | 1,377 (4.0) | 64 (2.0) |  |
| **Gender** |  |  |  |  |  |  |
| Male | 6.2 (0.4) | 571,113,588 | 12,431 (49.0) | 11,624 (49.1) | 807 (37.6) | **< 0.001** |
| Female | 9.6 (0.4) | 613,027,666 | 12,926 (51.0) | 11,539 (50.9) | 1,387 (62.4) |  |
| **Race/ethnicity** |  |  |  |  |  |  |
| Mexican American | 8.6 (0.7) | 89,101,932 | 3,674 (14.5) | 3,366 (7.5) | 308 (7.7) | **< 0.001** |
| Non-Hispanic Black | 9.8 (0.5) | 124,176,625 | 5,337 (21.1) | 4,859 (10.3) | 478 (13.2) |  |
| Non-Hispanic White | 7.4 (0.4) | 833,102,497 | 11,681 (46.1) | 10,696 (70.8) | 985 (64.7) |  |
| Other races | 9.8 (0.8) | 137,760,201 | 4,665 (18.4) | 4,242 (11.4) | 423 (14.5) |  |
| **BMI, kg/m^2^** | - | - | 29.12 ± 0.09 | 28.97 ± 0.09 | 30.88 ± 0.23 | **< 0.001** |
| **PIR** | - | - | 3.07 ± 0.04 | 3.15 ± 0.03 | 2.13 ± 0.07 | **< 0.001** |
| ≥ 300% | 4.2 (0.3) | 611,632,294 | 9,838 (38.8) | 9,444 (53.8) | 394 (26.3) | **< 0.001** |
| < 300% | 12.4 (0.5) | 572,508,960 | 15,519 (61.2) | 13,719 (46.2) | 1,800 (73.8) |  |
| **Education level** |  |  |  |  |  |  |
| Less than 9th grade | 13.2 (1.3) | 48,957,898 | 2,165 (8.5) | 1,893 (3.9) | 272 (6.7) | **< 0.001** |
| 9-11th grade (including 12th grade with no diploma) | 13.4 (0.9) | 114,617,982 | 3,384 (13.4) | 2,925 (9.1) | 459 (16.2) |  |
| High school grade/GED or equivalent | 9.7 (0.6) | 275,421,825 | 5,833 (23.0) | 5,292 (22.8) | 541 (28.2) |  |
| Some college or AA degree | 8.6 (0.5) | 380,180,823 | 7,733 (30.5) | 7,064 (31.9) | 669 (34.8) |  |
| College graduate or above | 3.6 (0.4) | 364,962,726 | 6,242 (24.6) | 5,989 (32.3) | 253 (14.1) |  |
| **Alcohol consumption** |  |  |  |  |  |  |
| Never | 6.5 (0.6) | 122,416,118 | 3,367 (13.3) | 3,120 (10.5) | 247 (8.2) | **< 0.001** |
| Former | 11.1 (0.9) | 160,448,474 | 4,239 (16.7) | 3,739 (13.0) | 500 (19.5) |  |
| Current | 7.5 (0.3) | 901,276,662 | 17,751 (70.0) | 16,304 (76.4) | 1,447 (72.3) |  |
| **Marital status** |  |  |  |  |  |  |
| Married/Living with partner | 5.9 (0.3) | 752,598,329 | 15,371 (60.6) | 14,360 (64.9) | 1,011 (47.7) | **< 0.001** |
| Never married | 10.4 (0.8) | 209,613,147 | 4,420 (17.4) | 3,978 (17.4) | 442 (21.8) |  |
| Widowed/Divorced/Separated | 14.9 (0.9) | 221,929,779 | 5,566 (22.0) | 4,825 (17.7) | 741 (30.6) |  |
| **Items of LE8 score (0-100)** |  |  |  |  |  |  |
| **Diet** | - | - | 39.44 ± 0.51 | 40.32 ± 0.51 | 29.21 ± 0.92 | **< 0.001** |
| Unfavorable score | 10.1 (0.5) | 599,051,916 | 12,782 (50.4) | 11,422 (49.3) | 1,360 (65.4) | **< 0.001** |
| Intermediate score | 6.3 (0.4) | 289,423,345 | 6,302 (24.9) | 5,819 (24.9) | 483 (19.2) |  |
| Favorable score | 5.0 (0.4) | 295,665,993 | 6,273 (24.7) | 5,922 (25.8) | 351 (15.5) |  |
| **PA** | - | - | 71.54 ± 0.50 | 72.67 ± 0.51 | 58.48 ± 1.43 | **< 0.001** |
| Unfavorable score | 12.3 (0.6) | 324,089,678 | 8,083 (31.9) | 7,136 (26.2) | 947 (40.9) | **< 0.001** |
| Intermediate score | 7.2 (1.2) | 59,532,580 | 1,206 (4.8) | 1,110 (5.1) | 96 (4.7) |  |
| Favorable score | 6.3 (0.3) | 800,518,996 | 16,068 (63.4) | 14,917 (68.7) | 1,151 (54.4) |  |
| **Nicotine exposure** | - | - | 71.10 ± 0.49 | 72.87 ± 0.45 | 50.53 ± 1.56 | **< 0.001** |
| Unfavorable score | 14.7 (0.7) | 265,696,864 | 5,779 (22.8) | 4,881 (20.6) | 898 (43.8) | **< 0.001** |
| Intermediate score | 6.5 (0.6) | 268,062,407 | 5,707 (22.5) | 5,284 (23.0) | 423 (18.6) |  |
| Favorable score | 5.4 (0.3) | 650,381,983 | 13,871 (54.7) | 12,998 (56.4) | 873 (37.6) |  |
| **Sleep health** | - | - | 83.35 ± 0.29 | 84.64 ± 0.26 | 68.30 ± 1.16 | **< 0.001** |
| Unfavorable score | 19.0 (0.9) | 175,478,530 | 4,467 (17.6) | 3,671 (13.0) | 796 (35.9) | **< 0.001** |
| Intermediate score | 7.5 (0.6) | 241,448,635 | 5,456 (21.5) | 4,992 (20.5) | 464 (19.3) |  |
| Favorable score | 5.6 (0.3) | 767,214,089 | 15,434 (60.9) | 14,500 (66.5) | 934 (44.9) |  |
| **BMI** | - | - | 60.08 ± 0.45 | 60.75 ± 0.46 | 52.24 ± 1.14 | **< 0.001** |
| Unfavorable score | 10.1 (0.4) | 448,070,779 | 9,935 (39.2) | 8,815 (36.9) | 1,120 (48.8) | **< 0.001** |
| Intermediate score | 6.5 (0.5) | 388,661,523 | 8,341 (32.9) | 7,775 (33.4) | 566 (26.5) |  |
| Favorable score | 6.7 (0.5) | 347,408,952 | 7,081 (27.9) | 6,573 (29.7) | 508 (24.8) |  |
| **Blood lipids (non–HDL-C)** | - | - | 63.49 ± 0.35 | 63.73 ± 0.37 | 60.68 ± 0.93 | **< 0.05** |
| Unfavorable score | 8.8 (0.4) | 440,283,865 | 9,330 (36.8) | 8,412 (36.8) | 918 (42.2) | **< 0.001** |
| Intermediate score | 6.9 (0.4) | 270,081,136 | 5,613 (22.1) | 5,167 (23.1) | 446 (19.9) |  |
| Favorable score | 7.7 (0.4) | 473,776,253 | 10,414 (41.1) | 9,584 (40.2) | 830 (38.0) |  |
| **Blood glucose** | - | - | 86.05 ± 0.25 | 86.40 ± 0.26 | 81.96 ± 0.83 | **< 0.001** |
| Unfavorable score | 11.7 (0.8) | 166,598,615 | 4,671 (18.4) | 4,106 (13.5) | 565 (20.2) | **< 0.001** |
| Intermediate score | 8.9 (0.8) | 201,806,038 | 5,019 (19.8) | 4,615 (17.0) | 404 (17.3) |  |
| Favorable score | 7.0 (0.4) | 815,736,601 | 15,667 (61.8) | 14,442 (69.4) | 1,225 (62.5) |  |
| **Blood pressure** | - | - | 69.03 ± 0.33 | 69.13 ± 0.35 | 67.82 ± 1.02 | 0.230 |
| Unfavorable score | 9.1 (0.7) | 249,775,713 | 6,307 (24.9) | 5,720 (20.9) | 587 (23.7) | 0.070 |
| Intermediate score | 7.3 (0.5) | 387,760,307 | 8,143 (32.1) | 7,516 (33.0) | 627 (29.6) |  |
| Favorable score | 7.8 (0.4) | 546,605,234 | 10,907 (43.0) | 9,927 (46.1) | 980 (46.7) |  |
| **Survey waves** |  |  |  |  |  |  |
| 2005-2006 | 5.9 (0.7) | 170,066,111 | 3,388 (13.4) | 3,175 (14.7) | 213 (10.8) | 0.120 |
| 2007-2008 | 8.6 (0.9) | 160,988,009 | 3,964 (15.6) | 3,591 (13.5) | 373 (14.8) |  |
| 2009-2010 | 8.0 (0.6) | 165,180,398 | 4,150 (16.4) | 3,764 (13.9) | 386 (14.1) |  |
| 2011-2012 | 8.2 (0.9) | 176,400,748 | 3,625 (14.3) | 3,301 (14.9) | 324 (15.3) |  |
| 2013-2014 | 9.1 (0.7) | 184,101,388 | 3,916 (15.4) | 3,539 (15.3) | 377 (18.0) |  |
| 2015-2016 | 7.8 (0.8) | 174,615,296 | 3,541 (14.0) | 3,249 (14.8) | 292 (14.4) |  |
| 2017-2018 | 7.7 (0.7) | 152,789,305 | 2,773 (10.9) | 2,544 (12.9) | 229 (12.6) |  |
| Footnotes: Continuous variables are presented as weighted mean ± SE, and categorical variables are presented as counting (n) and survey-weighted percentage (%). Score for items of LE8 was categorized into unfavorable score (0-49 points), intermediate score (50-79 points), and favorable score (80-100 points) according to AHA recommendation^[1]^. ^a^ Age-adjusted prevalence rates are present as [weighted number, % (SE)]. There were two steps to calculate them: First, the standard age proportions for age groups, based on the 2000 U.S Census Standard Population data, were calculated by dividing the age-specific Census population (P) by the total Census population number (T), and the standardizing proportions (P/T) should sum to 1. Second, the age-specific prevalence from the study population is multiplied by the proportion of people in that age group in the standard population, and results summed up to get the age-adjusted estimates. More detail can be got from: https://wwwn.cdc.gov/nchs/nhanes/tutorials/samplecode.aspx. ^b^ The *P*-values were assessed by t-test or Mann-Whitney U test (continuous variables) or by chi-square test or Fisher exact test (categorical variables) to represent the differences between participants with and without depressive symptom. *P*-values presented with bold valued were statistically significant. ^[1]^ Lloyd-Jones DM, Allen NB, Anderson CAM, et al. Life's Essential 8: Updating and Enhancing the American Heart Association's Construct of Cardiovascular Health: A Presidential Advisory From the American Heart Association. Circulation. 2022;146(5): e18-e43. doi:10.1161/CIR.0000000000001078 | | | | | | |
| Abbreviations: AA, Associate's degree; BMI, Body mass index; GED, General equivalent diploma; LE8, Life’s Essential 8; NHANES, National Health and Nutrition Examination Survey; Non-HDL-C, Non-high-density lipoprotein cholesterol; PA, Physical activity; PIR, Poverty-to-income ratio; SE, Standard error. | | | | | | |

| **Supplementary Table S6** Survey-weighted characteristic variables of the study participants stratified by survey waves, NHANES 2005-2018, U.S (n = 25,357) | | | | | | | | | | | |
| --- | --- | --- | --- | --- | --- | --- | --- | --- | --- | --- | --- |
| **Characteristic variables** | **Age-adjusted prevalence rate^a^** | **Estimate U.S population (n)** | **Total participants [n (%)]** | **Survey waves** | | | | | | | ***P*-value^b^** |
|  |  |  |  | **2005-2006** | **2007-2008** | **2009-2010** | **2011-2012** | **2013-2014** | **2015-2016** | **2017-2018** |  |
| **No. of participants** | 7.9 (0.3) | 1,184,141,254 | 25,357 (100.0) | 3,388 (13.4) | 3,964 (15.6) | 4,150 (16.4) | 3,625 (14.3) | 3,916 (15.4) | 3,541 (14.0) | 2,773 (10.9) | - |
| **Age, years** | - | - | 47.98 ± 0.26 | 47.55 ± 0.75 | 47.29 ± 0.53 | 47.81 ± 0.51 | 48.17 ± 0.95 | 48.47 ± 0.40 | 49.02 ± 0.74 | 47.38 ± 0.71 | 0.390 |
| 20-39 | 7.9 (0.4) | 408,025,337 | 8,203 (32.4) | 1,110 (35.1) | 1,206 (35.5) | 1,320 (34.2) | 1,247 (33.4) | 1,267 (33.1) | 1,127 (33.0) | 926 (37.4) | 0.300 |
| 40-59 | 9.3 (0.5) | 459,397,489 | 8,571 (33.8) | 1,157 (40.0) | 1,287 (40.0) | 1,428 (39.9) | 1,199 (39.4) | 1,369 (38.2) | 1,195 (37.6) | 936 (36.4) |  |
| 60-79 | 6.2 (0.5) | 271,522,095 | 7,142 (28.2) | 908 (21.5) | 1,229 (20.6) | 1,141 (21.7) | 988 (23.3) | 1,070 (24.7) | 1,017 (25.0) | 789 (23.4) |  |
| ≥ 80 | 4.1 (0.6) | 45,196,334 | 1,441 (5.7) | 213 (3.4) | 242 (3.87) | 261 (4.2) | 191 (4.0) | 210 (4.0) | 202 (4.4) | 122 (2.8) |  |
| **Gender** |  |  |  |  |  |  |  |  |  |  |  |
| Male | 6.2 (0.4) | 571,113,588 | 12,431 (49.0) | 1,723 (48.2) | 1,941 (46.6) | 2,030 (49.0) | 1,814 (49.0) | 1,873 (48.3) | 1,720 (48.4) | 1,330 (47.9) | 0.630 |
| Female | 9.6 (0.4) | 613,027,666 | 12,926 (51.0) | 1,665 (51.8) | 2,023 (53.5) | 2,120 (51.0) | 1,811 (51.0) | 2,043 (51.7) | 1,821 (51.6) | 1,443 (52.1) |  |
| **Race/ethnicity** |  |  |  |  |  |  |  |  |  |  |  |
| Mexican American | 8.6 (0.7) | 89,101,932 | 3,674 (14.5) | 617 (6.8) | 625 (7.4) | 698 (7.5) | 331 (7.0) | 483 (8.3) | 579 (7.8) | 341 (7.9) | 0.390 |
| Non-Hispanic Black | 9.8 (0.5) | 124,176,625 | 5,337 (21.1) | 747 (10.4) | 792 (10.5) | 701 (10.1) | 938 (10.8) | 776 (10.8) | 730 (10.0) | 653 (10.9) |  |
| Non-Hispanic White | 7.4 (0.4) | 833,102,497 | 11,681 (46.1) | 1,796 (75.4) | 2,020 (73.4) | 2,188 (72.6) | 1,486 (69.6) | 1,823 (68.2) | 1,341 (68.5) | 1,027 (64.9) |  |
| Other races | 9.8 (0.8) | 137,760,201 | 4,665 (18.4) | 228 (7.5) | 527 (8.7) | 563 (9.9) | 870 (12.7) | 834 (12.8) | 891 (13.8) | 752 (16.3) |  |
| **BMI, kg/m^2^** | - | - | 29.12 ± 0.09 | 28.68 ± 0.31 | 28.68 ± 0.17 | 28.93 ± 0.16 | 28.85 ± 0.23 | 29.24 ± 0.20 | 29.55 ± 0.29 | 29.94 ± 0.30 | **< 0.05** |
| **PIR** | - | - | 3.07 ± 0.04 | 3.17 ± 0.07 | 3.05 ± 0.10 | 3.08 ± 0.05 | 2.95 ± 0.11 | 2.98 ± 0.11 | 3.05 ± 0.10 | 3.22 ± 0.07 | 0.310 |
| ≥ 300% | 4.2 (0.3) | 611,632,294 | 9,838 (38.8) | 1,492 (54.8) | 1,454 (50.2) | 1,588 (53.0) | 1,342 (48.3) | 1,605 (50.6) | 1,222 (49.9) | 1,135 (55.5) | 0.400 |
| < 300% | 12.4 (0.5) | 572,508,960 | 15,519 (61.2) | 1,896 (45.2) | 2,510 (49.9) | 2,562 (47.0) | 2,283 (51.7) | 2,311 (49.4) | 2,319 (50.2) | 1,638 (44.7) |  |
| **Education level** |  |  |  |  |  |  |  |  |  |  |  |
| Less than 9th grade | 13.2 (1.3) | 48,957,898 | 2,165 (8.5) | 357 (4.9) | 450 (5.8) | 415 (4.7) | 261 (4.0) | 220 (3.5) | 316 (4.0) | 146 (2.1) | **< 0.001** |
| 9-11th grade (including 12th grade with no diploma) | 13.4 (0.9) | 114,617,982 | 3,384 (13.4) | 492 (10.2) | 688 (13.2) | 615 (11.5) | 477 (10.2) | 484 (9.7) | 369 (7.2) | 259 (5.6) |  |
| High school grade/GED or equivalent | 9.7 (0.6) | 275,421,825 | 5,833 (23.0) | 820 (25.1) | 980 (25.6) | 963 (22.7) | 747 (19.7) | 879 (21.7) | 819 (22.1) | 625 (26.6) |  |
| Some college or AA degree | 8.6 (0.5) | 380,180,823 | 7,733 (30.5) | 998 (32.1) | 1,047 (29.2) | 1,223 (31.3) | 1,138 (32.9) | 1,265 (33.7) | 1,080 (33.3) | 982 (31.9) |  |
| College graduate or above | 3.6 (0.4) | 364,962,726 | 6,242 (24.6) | 721 (27.7) | 799 (26.2) | 934 (29.8) | 1,002 (33.2) | 1,068 (31.4) | 957 (33.4) | 761 (33.8) |  |
| **Alcohol consumption** |  |  |  |  |  |  |  |  |  |  |  |
| Never | 6.5 (0.6) | 122,416,118 | 3,367 (13.3) | 408 (10.4) | 579 (11.7) | 485 (9.6) | 485 (9.7) | 546 (11.7) | 544 (11.2) | 320 (7.8) | **< 0.001** |
| Former | 11.1 (0.9) | 160,448,474 | 4,239 (16.7) | 732 (17.4) | 857 (17.8) | 760 (15.2) | 638 (14.2) | 678 (14.6) | 574 (14.4) | 0 (0.00) |  |
| Current | 7.5 (0.3) | 901,276,662 | 17,751 (70.0) | 2,248 (72.3) | 2,528 (70.5) | 2,905 (75.1) | 2,502 (76.1) | 2,692 (73.7) | 2,423 (74.4) | 2,453 (92.2) |  |
| **Marital status** |  |  |  |  |  |  |  |  |  |  |  |
| Married/Living with partner | 5.9 (0.3) | 752,598,329 | 15,371 (60.6) | 2,166 (66.3) | 2,426 (63.4) | 2,547 (64.2) | 2,049 (60.9) | 2,348 (62.0) | 2,153 (65.2) | 1,682 (63.2) | 0.300 |
| Never married | 10.4 (0.8) | 209,613,147 | 4,420 (17.4) | 488 (13.8) | 607 (17.7) | 688 (17.9) | 785 (20.1) | 719 (18.6) | 631 (16.8) | 502 (18.9) |  |
| Widowed/Divorced/Separated | 14.9 (0.9) | 221,929,779 | 5,566 (22.0) | 734 (19.9) | 931 (18.9) | 915 (17.8) | 791 (19.0) | 849 (19.4) | 757 (18.0) | 589 (17.9) |  |
| **Items of LE8 score (0-100)** |  |  |  |  |  |  |  |  |  |  |  |
| **Diet** | - | - | 39.44 ± 0.51 | 36.60 ± 0.98 | 37.57 ± 1.77 | 40.68 ± 0.69 | 42.62 ± 1.04 | 40.74 ± 0.98 | 39.62 ± 1.53 | 37.79 ± 1.92 | **< 0.05** |
| Unfavorable score | 10.1 (0.5) | 599,051,916 | 12,782 (50.4) | 1,832 (54.8) | 2,010 (53.3) | 2,089 (49.1) | 1,701 (46.3) | 1,906 (49.0) | 1,828 (50.1) | 1,416 (52.1) | 0.060 |
| Intermediate score | 6.3 (0.4) | 289,423,345 | 6,302 (24.9) | 844 (24.2) | 995 (23.4) | 1,057 (24.7) | 943 (25.7) | 954 (23.9) | 838 (24.1) | 671 (25.2) |  |
| Favorable score | 5.0 (0.4) | 295,665,993 | 6,273 (24.7) | 712 (21.0) | 959 (23.3) | 1,004 (26.1) | 981 (28.0) | 1,056 (27.1) | 875 (25.8) | 686 (22.8) |  |
| **PA** | - | - | 71.54 ± 0.50 | 57.14 ± 1.59 | 72.10 ± 1.52 | 73.26 ± 1.14 | 74.73 ± 1.53 | 71.39 ± 0.96 | 75.08 ± 1.26 | 77.61 ± 1.18 | **< 0.001** |
| Unfavorable score | 12.3 (0.6) | 324,089,678 | 8,083 (31.9) | 1,637 (43.5) | 1,279 (26.7) | 1,282 (25.4) | 1,005 (23.9) | 1,128 (27.1) | 1,058 (23.6) | 694 (21.0) | **< 0.001** |
| Intermediate score | 7.2 (1.2) | 59,532,580 | 1,206 (4.8) | 336 (11.0) | 154 (4.1) | 150 (3.6) | 168 (4.3) | 168 (4.3) | 130 (3.8) | 100 (4.1) |  |
| Favorable score | 6.3 (0.3) | 800,518,996 | 16,068 (63.4) | 1,415 (45.5) | 2,531 (69.3) | 2,718 (71.1) | 2,452 (71.8) | 2,620 (68.7) | 2,353 (72.6) | 1,979 (74.9) |  |
| **Nicotine exposure** | - | - | 71.10 ± 0.49 | 67.17 ± 0.93 | 68.01 ± 1.52 | 71.66 ± 1.27 | 71.07 ± 1.37 | 72.12 ± 1.34 | 72.69 ± 1.01 | 75.13 ± 1.59 | **< 0.001** |
| Unfavorable score | 14.7 (0.7) | 265,696,864 | 5,779 (22.8) | 823 (26.1) | 948 (25.3) | 966 (21.9) | 781 (22.1) | 879 (21.6) | 784 (21.0) | 598 (19.1) | **< 0.05** |
| Intermediate score | 6.5 (0.6) | 268,062,407 | 5,707 (22.5) | 834 (23.2) | 956 (22.7) | 967 (22.5) | 801 (23.5) | 833 (22.1) | 762 (23.6) | 554 (20.6) |  |
| Favorable score | 5.4 (0.3) | 650,381,983 | 13,871 (54.7) | 1,731 (50.7) | 2,060 (52.0) | 2,217 (55.6) | 2,043 (54.5) | 2,204 (56.3) | 1,995 (55.4) | 1,621 (60.3) |  |
| **Sleep health** | - | - | 83.35 ± 0.29 | 82.89 ± 0.71 | 81.72 ± 0.97 | 82.57 ± 0.46 | 82.61 ± 0.73 | 82.56 ± 0.62 | 86.65 ± 0.84 | 84.48 ± 0.80 | **< 0.001** |
| Unfavorable score | 19.0 (0.9) | 175,478,530 | 4,467 (17.6) | 596 (14.9) | 730 (16.1) | 733 (14.8) | 666 (14.9) | 666 (15.1) | 590 (13.1) | 486 (15.0) | **< 0.001** |
| Intermediate score | 7.5 (0.6) | 241,448,635 | 5,456 (21.5) | 778 (22.2) | 936 (22.7) | 995 (22.9) | 857 (23.7) | 956 (23.0) | 488 (12.4) | 446 (15.4) |  |
| Favorable score | 5.6 (0.3) | 767,214,089 | 15,434 (60.9) | 2,014 (62.9) | 2,298 (61.1) | 2,422 (62.3) | 2,102 (61.5) | 2,294 (61.9) | 2,463 (74.5) | 1,841 (69.6) |  |
| **BMI** | - | - | 60.08 ± 0.45 | 62.63 ± 1.52 | 62.64 ± 0.82 | 60.30 ± 0.90 | 61.75 ± 1.13 | 59.74 ± 0.86 | 57.88 ± 1.39 | 55.29 ± 1.48 | **< 0.001** |
| Unfavorable score | 10.1 (0.4) | 448,070,779 | 9,935 (39.2) | 1,218 (34.5) | 1,468 (34.2) | 1,641 (37.7) | 1,373 (35.6) | 1,517 (37.9) | 1,503 (40.6) | 1,215 (45.0) | **< 0.001** |
| Intermediate score | 6.5 (0.5) | 388,661,523 | 8,341 (32.9) | 1,166 (33.6) | 1,392 (34.7) | 1,387 (32.8) | 1,167 (33.7) | 1,262 (33.0) | 1,118 (32.5) | 849 (29.2) |  |
| Favorable score | 6.7 (0.5) | 347,408,952 | 7,081 (27.9) | 1,004 (32.0) | 1,104 (31.1) | 1,122 (29.5) | 1,085 (30.7) | 1,137 (29.2) | 920 (26.9) | 709 (25.8) |  |
| **Blood lipids (non–HDL-C)** | - | - | 63.49 ± 0.35 | 61.34 ± 0.83 | 60.62 ± 0.71 | 62.41 ± 0.72 | 61.78 ± 1.20 | 66.09 ± 0.62 | 65.06 ± 0.55 | 67.09 ± 1.35 | **< 0.001** |
| Unfavorable score | 8.8 (0.4) | 440,283,865 | 9,330 (36.8) | 1,287 (39.2) | 1,617 (41.6) | 1,639 (39.1) | 1,398 (40.7) | 1,280 (33.1) | 1,186 (34.1) | 923 (32.6) | **< 0.001** |
| Intermediate score | 6.9 (0.4) | 270,081,136 | 5,613 (22.1) | 793 (25.1) | 879 (22.2) | 914 (22.2) | 793 (22.6) | 885 (22.2) | 762 (23.1) | 587 (22.3) |  |
| Favorable score | 7.7 (0.4) | 473,776,253 | 10,414 (41.1) | 1,308 (35.6) | 1,468 (36.3) | 1,597 (38.7) | 1,434 (36.8) | 1,751 (44.7) | 1,593 (42.9) | 1,263 (45.1) |  |
| **Blood glucose** | - | - | 86.05 ± 0.25 | 89.70 ± 0.61 | 86.82 ± 0.70 | 86.38 ± 0.48 | 85.45 ± 0.62 | 85.95 ± 0.55 | 83.39 ± 0.95 | 84.70 ± 0.63 | **< 0.001** |
| Unfavorable score | 11.7 (0.8) | 166,598,615 | 4,671 (18.4) | 540 (12.3) | 739 (13.1) | 732 (12.8) | 672 (13.7) | 684 (14.2) | 747 (16.6) | 557 (15.8) | **< 0.001** |
| Intermediate score | 8.9 (0.8) | 201,806,038 | 5,019 (19.8) | 475 (11.5) | 806 (17.6) | 857 (18.8) | 754 (18.5) | 726 (16.0) | 777 (19.1) | 624 (18.0) |  |
| Favorable score | 7.0 (0.4) | 815,736,601 | 15,667 (61.8) | 2,373 (76.2) | 2,419 (69.3) | 2,561 (68.4) | 2,199 (67.9) | 2,506 (69.8) | 2,017 (64.3) | 1,592 (66.2) |  |
| **Blood pressure** | - | - | 69.03 ± 0.33 | 68.01 ± 0.72 | 69.71 ± 0.78 | 70.90 ± 1.01 | 68.84 ± 0.98 | 69.92 ± 0.69 | 67.66 ± 0.78 | 68.14 ± 1.09 | 0.080 |
| Unfavorable score | 9.1 (0.7) | 249,775,713 | 6,307 (24.9) | 861 (22.5) | 996 (20.6) | 944 (18.8) | 886 (21.0) | 940 (20.9) | 911 (21.7) | 769 (22.1) | 0.160 |
| Intermediate score | 7.3 (0.5) | 387,760,307 | 8,143 (32.1) | 1,096 (33.0) | 1,246 (31.9) | 1,290 (31.9) | 1,191 (34.1) | 1,199 (31.0) | 1,196 (33.7) | 925 (33.8) |  |
| Favorable score | 7.8 (0.4) | 546,605,234 | 10,907 (43.0) | 1,431 (44.5) | 1,722 (47.5) | 1,916 (49.3) | 1,548 (44.9) | 1,777 (48.1) | 1,434 (44.6) | 1,079 (44.1) |  |
| **Depressive symptom** |  |  |  |  |  |  |  |  |  |  |  |
| No | - | 1,090,357,765 | 23,163 (91.4) | 3,175 (94.0) | 3,591 (91.4) | 3,764 (92.0) | 3,301 (91.8) | 3,539 (90.8) | 3,249 (92.3) | 2,544 (92.3) | 0.120 |
| Yes | - | 93,783,490 | 2,194 (8.7) | 213 (6.0) | 373 (8.6) | 386 (8.0) | 324 (8.2) | 377 (9.2) | 292 (7.7) | 229 (7.7) |  |
| Footnotes: Continuous variables are presented as weighted mean ± SE, and categorical variables are presented as counting (n) and survey-weighted percentage (%). Score for items of LE8 was categorized into unfavorable score (0-49 points), intermediate score (50-79 points), and favorable score (80-100 points) according to AHA recommendation^[1]^. ^a^ Age-adjusted prevalence rates are present as [weighted number, % (SE)]. There were two steps to calculate them: First, the standard age proportions for age groups, based on the 2000 U.S Census Standard Population data, were calculated by dividing the age-specific Census population (P) by the total Census population number (T), and the standardizing proportions (P/T) should sum to 1. Second, the age-specific prevalence from the study population is multiplied by the proportion of people in that age group in the standard population, and results summed up to get the age-adjusted estimates. More detail can be got from: https://wwwn.cdc.gov/nchs/nhanes/tutorials/samplecode.aspx. ^b^ The *P*-values were assessed by one-way ANOVA (continuous variables) or by chi-square test (categorical variables) to represent the differences of participants in different survey waves. *P*-values presented with bold valued were statistically significant. ^[1]^ Lloyd-Jones DM, Allen NB, Anderson CAM, et al. Life's Essential 8: Updating and Enhancing the American Heart Association's Construct of Cardiovascular Health: A Presidential Advisory From the American Heart Association. Circulation. 2022;146(5): e18-e43. doi:10.1161/CIR.0000000000001078 | | | | | | | | | | | |
| Abbreviations: AA, Associate's degree; ANOVA, Analysis of variance; BMI, Body mass index; GED, General equivalent diploma; LE8, Life’s Essential 8; NHANES, National Health and Nutrition Examination Survey; Non-HDL-C, Non-high-density lipoprotein cholesterol; PA, Physical activity; PIR, Poverty-to-income ratio; SE, Standard error. | | | | | | | | | | | |

| **Supplementary Table S7** Association between total and individual LE8 score and risk of depressive symptom in survey-weighted logistic regression models stratified by gender, NHANES 2005-2018, U.S (n = 25,357) | | | | | | | | | | | | | | | | | | | |
| --- | --- | --- | --- | --- | --- | --- | --- | --- | --- | --- | --- | --- | --- | --- | --- | --- | --- | --- | --- |
| **LE8 variables** | **Male (n = 12,431)** | | | | | | | | |  | **Female (n = 12,926)** | | | | | | | | |
|  | **Cases/participants** | **Crude model** | |  | **Model 1** | |  | **Model 2** | |  | **Cases/participants** | **Crude model** | |  | **Model 1** | |  | **Model 2** | |
|  |  | **COR (95% CI)** | ***P*-value** |  | **AOR (95% CI)** | ***P*-value** |  | **AOR (95% CI)** | ***P*-value** |  |  | **COR (95% CI)** | ***P*-value** |  | **AOR (95% CI)** | ***P*-value** |  | **AOR (95% CI)** | ***P*-value** |
| **Total** |  |  |  |  |  |  |  |  |  |  |  |  |  |  |  |  |  |  |  |
| Unfavorable score | 259/1,962 | Reference | - |  | Reference | - |  | Reference | - |  | 458/2,081 | Reference | - |  | Reference | - |  | Reference | - |
| Intermediate score | 501/8,644 | **0.405(0.345,0.475)** | **< 0.001** |  | **0.386(0.328,0.454)** | **< 0.001** |  | **0.468(0.397,0.554)** | **< 0.001** |  | 813/8,121 | **0.394(0.348,0.448)** | **< 0.001** |  | **0.362(0.318,0.412)** | **< 0.001** |  | **0.445(0.389,0.510)** | **< 0.001** |
| Favorable score | 47/1,825 | **0.174(0.125,0.236)** | **< 0.001** |  | **0.155(0.111,0.212)** | **< 0.001** |  | **0.232(0.164,0.322)** | **< 0.001** |  | 116/2,724 | **0.158(0.127,0.194)** | **< 0.001** |  | **0.126(0.101,0.157)** | **< 0.001** |  | **0.200(0.158,0.252)** | **< 0.001** |
| *P* for trend | - | - | **< 0.001** |  | - | **< 0.001** |  | - | **< 0.001** |  | - | - | **< 0.001** |  | - | **< 0.001** |  | - | **< 0.001** |
| **Diet** |  |  |  |  |  |  |  |  |  |  |  |  |  |  |  |  |  |  |  |
| Unfavorable score | 528/6,728 | Reference | - |  | Reference | - |  | Reference | - |  | 832/6,054 | Reference | - |  | Reference | - |  | Reference | - |
| Intermediate score | 176/3,024 | **0.726(0.607,0.864)** | **< 0.001** |  | **0.725(0.606,0.864)** | **< 0.001** |  | 0.902(0.741,1.093) | 0.299 |  | 307/3,278 | **0.649(0.564,0.744)** | **< 0.001** |  | **0.656(0.569,0.753)** | **< 0.001** |  | 0.888(0.758,1.037) | 0.136 |
| Favorable score | 103/2,679 | **0.470(0.376,0.580)** | **< 0.001** |  | **0.470(0.375,0.582)** | **< 0.001** |  | **0.766(0.596,0.974)** | **< 0.05** |  | 248/3,594 | **0.465(0.400,0.539)** | **< 0.001** |  | **0.468(0.402,0.544)** | **< 0.001** |  | **0.756(0.635,0.897)** | **< 0.05** |
| *P* for trend | - | - | **< 0.001** |  | - | **< 0.001** |  | - | **< 0.05** |  | - | - | **< 0.001** |  | - | **< 0.001** |  | - | **< 0.001** |
| **PA** |  |  |  |  |  |  |  |  |  |  |  |  |  |  |  |  |  |  |  |
| Unfavorable score | 301/3,383 | Reference | - |  | Reference | - |  | Reference | - |  | 646/4,700 | Reference | - |  | Reference | - |  | Reference | - |
| Intermediate score | 33/521 | 0.692(0.469,0.989) | 0.053 |  | **0.645(0.436,0.923)** | **< 0.05** |  | 0.784(0.510,1.165) | 0.247 |  | 63/685 | **0.636(0.480,0.828)** | **< 0.001** |  | **0.592(0.446,0.772)** | **< 0.001** |  | **0.623(0.451,0.844)** | **< 0.05** |
| Favorable score | 473/8,527 | **0.601(0.518,0.699)** | **< 0.001** |  | **0.557(0.478,0.650)** | **< 0.001** |  | **0.686(0.577,0.815)** | **< 0.001** |  | 678/7,541 | **0.620(0.553,0.695)** | **< 0.001** |  | **0.573(0.509,0.644)** | **< 0.001** |  | **0.709(0.621,0.810)** | **< 0.001** |
| *P* for trend | - | - | **< 0.001** |  | - | **< 0.001** |  | - | **< 0.001** |  | - | - | **< 0.001** |  | - | **< 0.001** |  | - | **< 0.001** |
| **Nicotine exposure** |  |  |  |  |  |  |  |  |  |  |  |  |  |  |  |  |  |  |  |
| Unfavorable score | 362/3,310 | Reference | - |  | Reference | - |  | Reference | - |  | 536/2,469 | Reference | - |  | Reference | - |  | Reference | - |
| Intermediate score | 201/3,411 | **0.510(0.426,0.609)** | **< 0.001** |  | **0.540(0.446,0.652)** | **< 0.001** |  | **0.724(0.585,0.895)** | **< 0.05** |  | 222/2,296 | **0.386(0.326,0.456)** | **< 0.001** |  | **0.404(0.339,0.479)** | **< 0.001** |  | **0.581(0.476,0.707)** | **< 0.001** |
| Favorable score | 244/5,710 | **0.364(0.307,0.430)** | **< 0.001** |  | **0.368(0.310,0.435)** | **< 0.001** |  | **0.551(0.453,0.670)** | **< 0.001** |  | 629/8,161 | **0.301(0.266,0.342)** | **< 0.001** |  | **0.297(0.261,0.339)** | **< 0.001** |  | **0.481(0.412,0.562)** | **< 0.001** |
| *P* for trend | - | - | **< 0.001** |  | - | **< 0.001** |  | - | **< 0.001** |  | - | - | **< 0.001** |  | - | **< 0.001** |  | - | **< 0.001** |
| **Sleep health** |  |  |  |  |  |  |  |  |  |  |  |  |  |  |  |  |  |  |  |
| Unfavorable score | 287/2,176 | Reference | - |  | Reference | - |  | Reference | - |  | 509/2,291 | Reference | - |  | Reference | - |  | Reference | - |
| Intermediate score | 180/2,828 | **0.447(0.367,0.543)** | **< 0.001** |  | **0.430(0.353,0.523)** | **< 0.001** |  | **0.562(0.453,0.695)** | **< 0.001** |  | 284/2,628 | **0.424(0.362,0.496)** | **< 0.001** |  | **0.412(0.352,0.483)** | **< 0.001** |  | **0.467(0.391,0.557)** | **< 0.001** |
| Favorable score | 340/7,427 | **0.316(0.268,0.373)** | **< 0.001** |  | **0.308(0.261,0.365)** | **< 0.001** |  | **0.405(0.338,0.487)** | **< 0.001** |  | 594/8,007 | **0.281(0.247,0.319)** | **< 0.001** |  | **0.275(0.241,0.313)** | **< 0.001** |  | **0.368(0.319,0.425)** | **< 0.001** |
| *P* for trend | - | - | **< 0.001** |  | - | **< 0.001** |  | - | **< 0.001** |  | - | - | **< 0.001** |  | - | **< 0.001** |  | - | **< 0.001** |
| **BMI** |  |  |  |  |  |  |  |  |  |  |  |  |  |  |  |  |  |  |  |
| Unfavorable score | 353/4,450 | Reference | - |  | Reference | - |  | Reference | - |  | 767/5,485 | Reference | - |  | Reference | - |  | Reference | - |
| Intermediate score | 241/4,720 | **0.624(0.527,0.739)** | **< 0.001** |  | **0.634(0.534,0.751)** | **< 0.001** |  | **0.694(0.573,0.838)** | **< 0.001** |  | 325/3,621 | **0.607(0.528,0.695)** | **< 0.001** |  | **0.619(0.538,0.710)** | **< 0.001** |  | **0.727(0.622,0.849)** | **< 0.001** |
| Favorable score | 213/3,261 | **0.811(0.679,0.967)** | **< 0.05** |  | **0.831(0.694,0.993)** | **< 0.05** |  | **0.780(0.632,0.962)** | **< 0.05** |  | 295/3,820 | **0.515(0.446,0.592)** | **< 0.001** |  | **0.509(0.440,0.588)** | **< 0.001** |  | **0.683(0.577,0.807)** | **< 0.001** |
| *P* for trend | - | - | **< 0.05** |  | - | **< 0.05** |  | - | **< 0.05** |  | - | - | **< 0.001** |  | - | **< 0.001** |  | - | **< 0.001** |
| **Blood lipids (non–HDL-C)** | |  |  |  |  |  |  |  |  |  |  |  |  |  |  |  |  |  |  |
| Unfavorable score | 344/4,783 | Reference | - |  | Reference | - |  | Reference | - |  | 574/4,547 | Reference | - |  | Reference | - |  | Reference | - |
| Intermediate score | 156/2,730 | **0.782(0.642,0.949)** | **< 0.05** |  | **0.780(0.640,0.947)** | **< 0.05** |  | 0.840(0.677,1.038) | 0.110 |  | 290/2,883 | **0.774(0.666,0.898)** | **< 0.001** |  | **0.738(0.634,0.859)** | **< 0.001** |  | 0.901(0.760,1.066) | 0.227 |
| Favorable score | 307/4,918 | 0.859(0.732,1.007) | 0.062 |  | 0.910(0.773,1.070) | 0.253 |  | 0.939(0.784,1.124) | 0.491 |  | 523/5,496 | **0.728(0.642,0.825)** | **< 0.001** |  | **0.719(0.631,0.819)** | **< 0.001** |  | 0.896(0.773,1.038) | 0.142 |
| *P* for trend | - | - | 0.059 |  | - | 0.229 |  | - | 0.487 |  | - | - | **< 0.001** |  | - | **< 0.001** |  | - | 0.149 |
| **Blood glucose** |  |  |  |  |  |  |  |  |  |  |  |  |  |  |  |  |  |  |  |
| Unfavorable score | 215/2,387 | Reference | - |  | Reference | - |  | Reference | - |  | 350/2,284 | Reference | - |  | Reference | - |  | Reference | - |
| Intermediate score | 144/2,547 | **0.605(0.486,0.753)** | **< 0.001** |  | **0.591(0.474,0.736)** | **< 0.001** |  | **0.595(0.467,0.758)** | **< 0.001** |  | 260/2,472 | **0.649(0.547,0.771)** | **< 0.001** |  | **0.638(0.536,0.758)** | **< 0.001** |  | **0.681(0.560,0.827)** | **< 0.001** |
| Favorable score | 448/7,497 | **0.642(0.543,0.762)** | **< 0.001** |  | **0.576(0.480,0.692)** | **< 0.001** |  | **0.685(0.552,0.853)** | **< 0.001** |  | 777/8,170 | **0.581(0.507,0.666)** | **< 0.001** |  | **0.511(0.442,0.591)** | **< 0.001** |  | **0.671(0.563,0.801)** | **< 0.001** |
| *P* for trend | - | - | **< 0.001** |  | - | **< 0.001** |  | - | **< 0.05** |  | - | - | **< 0.001** |  | - | **< 0.001** |  | - | **< 0.001** |
| **Blood pressure** |  |  |  |  |  |  |  |  |  |  |  |  |  |  |  |  |  |  |  |
| Unfavorable score | 212/3,101 | Reference | - |  | Reference | - |  | Reference | - |  | 375/3,206 | Reference | - |  | Reference | - |  | Reference | - |
| Intermediate score | 288/4,705 | 0.889(0.740,1.068) | 0.207 |  | **0.823(0.680,0.998)** | **< 0.05** |  | 0.890(0.720,1.101) | 0.280 |  | 339/3,438 | **0.826(0.707,0.965)** | **< 0.05** |  | **0.716(0.609,0.841)** | **< 0.001** |  | 0.843(0.705,1.007) | 0.059 |
| Favorable score | 307/4,625 | 0.969(0.809,1.163) | 0.732 |  | 0.898(0.740,1.090) | 0.273 |  | 1.045(0.844,1.294) | 0.688 |  | 673/6,282 | 0.906(0.793,1.037) | 0.148 |  | **0.766(0.658,0.892)** | **< 0.001** |  | 0.999(0.844,1.184) | 0.991 |
| *P* for trend | - | - | 0.854 |  | - | 0.423 |  | - | 0.491 |  | - | - | 0.268 |  | - | **< 0.05** |  | - | 0.740 |
| Footnotes: The total LE8 score was calculated as the mean of the sum of all 8 items of LE8 score and similarly ranged from 0 (if the mean score of all items was 0) to 100 (optimal CVH). Score for total LE8 and its items was categorized into unfavorable score (0-49 points), intermediate score (50-79 points), and favorable score (80-100 points) according to AHA recommendation^[1]^. For each sub-item of LE8 score: Crude model was unadjusted. Model 1 was adjusted for age, gender, and race/ethnicity. Model 2 was adjusted for age, gender, race/ethnicity, PIR, education level, alcohol consumption, marital status. In the case of total LE8 score, Model 2 was adjusted for age, gender, race/ethnicity, PIR, education level, alcohol consumption, and marital status. Results of COR (95% CI), AOR (95% CI), *P* for tend, and *P*-value presented with bold valued were statistically significant with *P*-value < 0.05 or *P*-value < 0.001. ^[1]^ Lloyd-Jones DM, Allen NB, Anderson CAM, et al. Life's Essential 8: Updating and Enhancing the American Heart Association's Construct of Cardiovascular Health: A Presidential Advisory From the American Heart Association. Circulation. 2022;146(5):e18-e43. doi:10.1161/CIR.0000000000001078 | | | | | | | | | | | | | | | | | | | |
| Abbreviations: AHA, American Heart Association; AOR, Adjusted odds ratio; BMI, Body mass index; CI, Confidence interval; COR, Crude odds ratio; CVH, Cardiovascular health; LE8, Life’s Essential 8; NHANES, National Health and Nutrition Examination Survey; Non-HDL-C, Non-high-density lipoprotein cholesterol; PA, Physical activity; PIR, Poverty-to-income ratio. | | | | | | | | | | | | | | | | | | | |

| **Supplementary Table S8** Association between total and individual LE8 score and risk of depressive symptom in survey-weighted logistic regression models stratified by race/ethnicity, NHANES 2005-2018, U.S (n = 25,357) | | | | | | | | | | | | | | | |
| --- | --- | --- | --- | --- | --- | --- | --- | --- | --- | --- | --- | --- | --- | --- | --- |
| **LE8 variables** | **Mexican American (n = 3,674)** | | |  | **Non-Hispanic Black (n = 5,337)** | | |  | **Non-Hispanic White (n = 11,681)** | | |  | **Other races (n = 4,665)** | | |
|  | **Cases/participants** | **COR (95% CI)** | **AOR (95% CI)** |  | **Cases/participants** | **COR (95% CI)** | **AOR (95% CI)** |  | **Cases/participants** | **COR (95% CI)** | **AOR (95% CI)** |  | **Cases/participants** | **COR (95% CI)** | **AOR (95% CI)** |
| **Total** |  |  |  |  |  |  |  |  |  |  |  |  |  |  |  |
| Unfavorable score | 76/525 | Reference | Reference |  | 180/1,252 | Reference | Reference |  | 351/1,728 | Reference | Reference |  | 110/538 | Reference | Reference |
| Intermediate score | 201/2,535 | **0.509 (0.385,0.678)^**^** | **0.573 (0.428,0.772)^**^** |  | 269/3,534 | **0.491 (0.402,0.601)^**^** | **0.515 (0.416,0.638)^**^** |  | 573/7,737 | **0.314 (0.272,0.363)^**^** | **0.413 (0.354,0.482)^**^** |  | 271/2,959 | **0.392 (0.308,0.502)^**^** | **0.458 (0.355,0.595)^**^** |
| Favorable score | 31/614 | **0.314 (0.201,0.481)^**^** | **0.354 (0.219,0.562)^**^** |  | 29/551 | **0.331 (0.216,0.489)^**^** | **0.337 (0.215,0.512)^**^** |  | 61/2,216 | **0.111 (0.083,0.146)^**^** | **0.174 (0.127,0.233)^**^** |  | 42/1,168 | **0.145 (0.099,0.209)^**^** | **0.195 (0.129,0.290)^**^** |
| *P* for trend | - | **< 0.001** | **< 0.001** |  | - | **< 0.001** | **< 0.001** |  | - | **< 0.001** | **< 0.001** |  | - | **< 0.001** | **< 0.001** |
| **Diet** |  |  |  |  |  |  |  |  |  |  |  |  |  |  |  |
| Unfavorable score | 156/1,805 | Reference | Reference |  | 310/2,998 | Reference | Reference |  | 667/6,021 | Reference | Reference |  | 227/1,958 | Reference | Reference |
| Intermediate score | 86/1,036 | 0.957 (0.724,1.257) | 0.989 (0.727,1.336) |  | 112/1,277 | 0.834 (0.662,1.043) | 0.942 (0.723,1.220) |  | 185/2,799 | **0.568 (0.478,0.672)^**^** | 0.828 (0.684,0.999) |  | 100/1,190 | **0.700 (0.544,0.893)^*^** | 0.941 (0.714,1.235) |
| Favorable score | 66/833 | 0.910 (0.670,1.222) | 0.902 (0.634,1.271) |  | 56/1,062 | **0.483 (0.357,0.642)^**^** | **0.690 (0.491,0.953)^*^** |  | 133/2,861 | **0.391 (0.322,0.472)^**^** | **0.739 (0.591,0.919)^*^** |  | 96/1,517 | **0.515 (0.400,0.658)^**^** | 0.773 (0.574,1.034) |
| *P* for trend | - | 0.529 | 0.593 |  | - | **< 0.001** | **< 0.05** |  | - | **< 0.001** | **< 0.05** |  | - | **< 0.001** | 0.096 |
| **PA** |  |  |  |  |  |  |  |  |  |  |  |  |  |  |  |
| Unfavorable score | 135/1,333 | Reference | Reference |  | 211/1,887 | Reference | Reference |  | 441/3,501 | Reference | Reference |  | 160/1,362 | Reference | Reference |
| Intermediate score | 10/167 | 0.565 (0.273,1.045) | 0.552 (0.251,1.079) |  | 21/251 | 0.725 (0.441,1.133) | 0.639 (0.347,1.099) |  | 46/587 | **0.590 (0.424,0.802)^**^** | **0.653 (0.449,0.928)^*^** |  | 19/201 | 0.784 (0.462,1.263) | 0.925 (0.516,1.577) |
| Favorable score | 163/2,174 | **0.719 (0.567,0.914)^*^** | 0.881 (0.671,1.159) |  | 246/3,199 | **0.662 (0.545,0.803)^**^** | **0.758 (0.604,0.953)^*^** |  | 498/7,593 | **0.487 (0.426,0.558)^**^** | **0.599 (0.511,0.702)^**^** |  | 244/3,102 | **0.641 (0.520,0.793)^**^** | 0.800 (0.630,1.021) |
| *P* for trend | - | **< 0.05** | 0.386 |  | - | **< 0.001** | **< 0.05** |  | - | **< 0.001** | **< 0.001** |  | - | **< 0.001** | 0.070 |
| **Nicotine exposure** |  |  |  |  |  |  |  |  |  |  |  |  |  |  |  |
| Unfavorable score | 80/633 | Reference | Reference |  | 197/1,400 | Reference | Reference |  | 465/2,930 | Reference | Reference |  | 156/816 | Reference | Reference |
| Intermediate score | 54/735 | **0.548 (0.380,0.786)^**^** | **0.525 (0.346,0.789)^*^** |  | 79/921 | **0.573 (0.433,0.752)^**^** | 0.870 (0.623,1.205) |  | 209/3,196 | **0.371 (0.312,0.440)^**^** | **0.681 (0.553,0.836)^**^** |  | 81/855 | **0.443 (0.331,0.589)^**^** | **0.532 (0.378,0.745)^**^** |
| Favorable score | 174/2,306 | **0.564 (0.427,0.750)^**^** | **0.525 (0.377,0.734)^**^** |  | 202/3,016 | **0.438 (0.356,0.540)^**^** | **0.496 (0.384,0.640)^**^** |  | 311/5,555 | **0.314 (0.270,0.366)^**^** | **0.607 (0.504,0.731)^**^** |  | 186/2,994 | **0.280 (0.223,0.353)^**^** | **0.365 (0.277,0.482)^**^** |
| *P* for trend | - | **< 0.001** | **< 0.001** |  | - | **< 0.001** | **< 0.001** |  | - | **< 0.001** | **< 0.001** |  | - | **< 0.001** | **< 0.001** |
| **Sleep health** |  |  |  |  |  |  |  |  |  |  |  |  |  |  |  |
| Unfavorable score | 95/556 | Reference | Reference |  | 218/1,405 | Reference | Reference |  | 339/1,740 | Reference | Reference |  | 144/766 | Reference | Reference |
| Intermediate score | 61/774 | **0.415 (0.294,0.583)^**^** | **0.465 (0.318,0.676)^**^** |  | 97/1,371 | **0.415 (0.321,0.532)^**^** | **0.444 (0.334,0.587)^**^** |  | 213/2,282 | **0.425 (0.353,0.511)^**^** | **0.584 (0.475,0.717)^**^** |  | 93/1,029 | **0.429 (0.324,0.567)^**^** | **0.457 (0.334,0.623)^**^** |
| Favorable score | 152/2,344 | **0.336 (0.256,0.444)^**^** | **0.388 (0.287,0.527)^**^** |  | 163/2,561 | **0.370 (0.298,0.458)^**^** | **0.397 (0.313,0.503)^**^** |  | 433/7,659 | **0.248 (0.213,0.289)^**^** | **0.397 (0.335,0.472)^**^** |  | 186/2,870 | **0.299 (0.237,0.379)^**^** | **0.372 (0.287,0.483)^**^** |
| *P* for trend | - | **< 0.001** | **< 0.001** |  | - | **< 0.001** | **< 0.001** |  | - | **< 0.001** | **< 0.001** |  | - | **< 0.001** | **< 0.001** |
| **BMI** |  |  |  |  |  |  |  |  |  |  |  |  |  |  |  |
| Unfavorable score | 170/1,610 | Reference | Reference |  | 257/2,572 | Reference | Reference |  | 489/4,314 | Reference | Reference |  | 204/1,439 | Reference | Reference |
| Intermediate score | 87/1,396 | **0.563 (0.429,0.735)^**^** | **0.668 (0.494,0.897)^*^** |  | 114/1,519 | **0.731 (0.579,0.918)^*^** | 0.894 (0.683,1.164) |  | 252/3,882 | **0.543 (0.463,0.636)^**^** | **0.712 (0.593,0.853)^**^** |  | 113/1,544 | **0.478 (0.374,0.608)^**^** | **0.625 (0.474,0.822)^**^** |
| Favorable score | 51/668 | **0.700 (0.501,0.964)^*^** | 0.847 (0.580,1.217) |  | 107/1,246 | 0.846 (0.666,1.069) | 1.008 (0.757,1.337) |  | 244/3,485 | **0.589 (0.501,0.691)^**^** | **0.701 (0.577,0.850)^**^** |  | 106/1,682 | **0.407 (0.317,0.520)^**^** | **0.558 (0.417,0.743)^**^** |
| *P* for trend | - | **< 0.001** | 0.109 |  | - | 0.064 | 0.912 |  | - | **< 0.001** | **< 0.001** |  | - | **< 0.001** | **< 0.001** |
| **Blood lipids (non–HDL-C)** | |  |  |  |  |  |  |  |  |  |  |  |  |  |  |
| Unfavorable score | 127/1,425 | Reference | Reference |  | 161/1,768 | Reference | Reference |  | 439/4,384 | Reference | Reference |  | 191/1,753 | Reference | Reference |
| Intermediate score | 76/920 | 0.920 (0.681,1.236) | 0.934 (0.663,1.308) |  | 93/1,086 | 0.935 (0.713,1.218) | 0.918 (0.677,1.240) |  | 194/2,532 | **0.746 (0.624,0.888)^**^** | 0.922 (0.756,1.121) |  | 83/1,075 | **0.684 (0.520,0.893)^*^** | 0.748 (0.551,1.008) |
| Favorable score | 105/1,329 | 0.877 (0.668,1.148) | 0.947 (0.698,1.285) |  | 224/2,483 | 0.990 (0.801,1.225) | 0.943 (0.735,1.213) |  | 352/4,765 | **0.717 (0.619,0.830)^**^** | 0.942 (0.796,1.116) |  | 149/1,837 | **0.722 (0.576,0.903)^*^** | 0.833 (0.642,1.081) |
| *P* for trend | - | 0.337 | 0.729 |  | - | 0.955 | 0.680 |  | - | **< 0.001** | 0.487 |  | - | **< 0.05** | 0.165 |
| **Blood glucose** |  |  |  |  |  |  |  |  |  |  |  |  |  |  |  |
| Unfavorable score | 100/769 | Reference | Reference |  |  | Reference | Reference |  | 228/1,726 | Reference | Reference |  | 101/852 | Reference | Reference |
| Intermediate score | 44/711 | **0.441 (0.302,0.635)^**^** | **0.488 (0.325,0.723)^**^** |  | 136/1,324 | 0.794 (0.607,1.036) | **0.644 (0.473,0.875)^*^** |  | 177/2,100 | **0.605 (0.491,0.744)^**^** | **0.657 (0.517,0.835)^**^** |  | 78/948 | **0.667 (0.487,0.909)^*^** | **0.686 (0.484,0.971)^*^** |
| Favorable score | 164/2,194 | **0.540 (0.416,0.705)^**^** | 0.751 (0.541,1.046) |  | 105/1,260 | 0.823 (0.660,1.029) | **0.602 (0.450,0.808)^**^** |  | 580/7,855 | **0.524 (0.446,0.617)^**^** | **0.623 (0.504,0.773)^**^** |  | 244/2,865 | **0.692 (0.543,0.888)^*^** | 0.839 (0.614,1.153) |
| *P* for trend | - | **< 0.001** | 0.164 |  | 237/2,753 | 0.120 | **< 0.001** |  | - | **< 0.001** | **< 0.001** |  | - | **< 0.05** | 0.460 |
| **Blood pressure** |  |  |  |  |  |  |  |  |  |  |  |  |  |  |  |
| Unfavorable score | 75/698 | Reference | Reference |  | 170/1,744 | Reference | Reference |  | 245/2,902 | Reference | Reference |  | 97/963 | Reference | Reference |
| Intermediate score | 92/1,241 | **0.665 (0.483,0.918)^*^** | 0.791 (0.548,1.146) |  | 126/1,720 | **0.732 (0.574,0.930)^*^** | 0.776 (0.586,1.024) |  | 286/3,708 | 0.906 (0.759,1.083) | 0.929 (0.756,1.142) |  | 123/1,474 | 0.813 (0.615,1.077) | 0.854 (0.617,1.186) |
| Favorable score | 141/1,735 | **0.735 (0.549,0.991)^*^** | 0.960 (0.672,1.382) |  | 182/1,873 | 0.997 (0.800,1.242) | 0.954 (0.725,1.256) |  | 454/5,071 | 1.066 (0.907,1.256) | 1.076 (0.882,1.314) |  | 203/2,228 | 0.895 (0.696,1.158) | 0.995 (0.728,1.366) |
| *P* for trend | - | 0.103 | 0.939 |  | - | 0.979 | 0.818 |  | - | 0.280 | 0.328 |  | - | 0.570 | 0.809 |
| Footnotes: The total LE8 score was calculated as the mean of the sum of all 8 items of LE8 score and similarly ranged from 0 (if the mean score of all items was 0) to 100 (optimal CVH). Score for total LE8 and its items was categorized into unfavorable score (0-49 points), intermediate score (50-79 points), and favorable score (80-100 points) according to AHA recommendation^[1]^. For each sub-item of LE8 score, crude model was unadjusted, multivariate model was adjusted for age, gender, race/ethnicity, PIR, education level, alcohol consumption, marital status, multivariate model was adjusted for age, gender, race/ethnicity, PIR, education level, alcohol consumption, and marital status. Results of COR (95% CI), AOR (95% CI), and *P* for tend presented with bold valued were statistically significant with *P*-value < 0.05 or *P*-value < 0.001.  ^*^ *P*-value < 0.05, ^**^ *P*-value < 0.001. ^[1]^ Lloyd-Jones DM, Allen NB, Anderson CAM, et al. Life's Essential 8: Updating and Enhancing the American Heart Association's Construct of Cardiovascular Health: A Presidential Advisory From the American Heart Association. Circulation. 2022;146(5):e18-e43. doi:10.1161/CIR.0000000000001078 | | | | | | | | | | | | | | | |
| Abbreviations: AHA, American Heart Association; AOR, Adjusted odds ratio; BMI, Body mass index; CI, Confidence interval; COR, Crude odds ratio; CVH, Cardiovascular health; LE8, Life’s Essential 8; NHANES, National Health and Nutrition Examination Survey; Non-HDL-C, Non-high-density lipoprotein cholesterol; PA, Physical activity; PIR, Poverty-to-income ratio. | | | | | | | | | | | | | | | |

| **Supplementary Table S9** Association between total and individual LE8 score and risk of depressive symptom in survey-weighted logistic regression models stratified by survey wave groups, NHANES 2005-2018, U.S (n = 25,357) | | | | | | | | | | | | | | | | | | | |
| --- | --- | --- | --- | --- | --- | --- | --- | --- | --- | --- | --- | --- | --- | --- | --- | --- | --- | --- | --- |
| **LE8 variables** | **Survey waves, 2005-2010** | | | | | | | | |  | **Survey waves, 2011-2018** | | | | | | | | |
|  | **Cases/participants** | **Crude model** | |  | **Model 1** | |  | **Model 2** | |  | **Cases/participants** | **Crude model** | |  | **Model 1** | |  | **Model 2** | |
|  |  | **COR (95% CI)** | ***P*-value** |  | **AOR (95% CI)** | ***P*-value** |  | **AOR (95% CI)** | ***P*-value** |  |  | **COR (95% CI)** | ***P*-value** |  | **AOR (95% CI)** | ***P*-value** |  | **AOR (95% CI)** | ***P*-value** |
| **Total** |  |  |  |  |  |  |  |  |  |  |  |  |  |  |  |  |  |  |  |
| Unfavorable score | 326/1,918 | Reference | - |  | Reference | - |  | Reference | - |  | 391/2,125 | Reference | - |  | Reference | - |  | Reference | - |
| Intermediate score | 587/7,769 | **0.399 (0.345,0.462)** | **< 0.001** |  | **0.370 (0.318,0.431)** | **< 0.001** |  | **0.453 (0.388,0.530)** | **< 0.001** |  | 727/8,996 | **0.390 (0.341,0.446)** | **< 0.001** |  | **0.375 (0.327,0.430)** | **< 0.001** |  | **0.462 (0.401,0.532)** | **< 0.001** |
| Favorable score | 59/1,815 | **0.164 (0.122,0.217)** | **< 0.001** |  | **0.124 (0.092,0.166)** | **< 0.001** |  | **0.190 (0.139,0.256)** | **< 0.001** |  | 104/2,734 | **0.175 (0.139,0.219)** | **< 0.001** |  | **0.144 (0.114,0.182)** | **< 0.001** |  | **0.234 (0.182,0.297)** | **< 0.001** |
| *P* for trend | - | - | **< 0.001** |  | - | **< 0.001** |  | - | **< 0.001** |  | - | - | **< 0.001** |  | - | **< 0.001** |  | - | **< 0.001** |
| **Diet** |  |  |  |  |  |  |  |  |  |  |  |  |  |  |  |  |  |  |  |
| Unfavorable score | 623/5,931 | Reference | - |  | Reference | - |  | Reference | - |  | 737/6,851 | Reference | - |  | Reference | - |  | Reference | - |
| Intermediate score | 200/2,896 | **0.632 (0.534,0.745)** | **< 0.001** |  | **0.620 (0.523,0.733)** | **< 0.001** |  | **0.794 (0.656,0.958)** | **< 0.05** |  | 283/3,406 | **0.752 (0.650,0.867)** | **< 0.001** |  | **0.728 (0.628,0.840)** | **< 0.001** |  | 0.959 (0.817,1.124) | 0.609 |
| Favorable score | 149/2,675 | **0.503 (0.416,0.603)** | **< 0.001** |  | **0.491 (0.405,0.592)** | **< 0.001** |  | **0.780 (0.628,0.964)** | **< 0.05** |  | 202/3,598 | **0.493 (0.419,0.579)** | **< 0.001** |  | **0.460 (0.389,0.541)** | **< 0.001** |  | **0.744 (0.615,0.896)** | **< 0.05** |
| *P* for trend | - | - | **< 0.001** |  | - | **< 0.001** |  | - | **< 0.05** |  | - | - | **< 0.001** |  | - | **< 0.001** |  | - | **< 0.05** |
| **PA** |  |  |  |  |  |  |  |  |  |  |  |  |  |  |  |  |  |  |  |
| Unfavorable score | 467/4,198 | Reference | - |  | Reference | - |  | Reference | - |  | 480/3,885 | Reference | - |  | Reference | - |  | Reference | - |
| Intermediate score | 45/640 | **0.604 (0.434,0.821)** | **< 0.05** |  | **0.558 (0.400,0.760)** | **< 0.001** |  | **0.583 (0.400,0.827)** | **< 0.05** |  | 51/566 | **0.702 (0.513,0.942)** | **< 0.05** |  | **0.668 (0.488,0.898)** | **< 0.05** |  | 0.794 (0.555,1.109) | 0.190 |
| Favorable score | 460/6,664 | **0.592 (0.518,0.678)** | **< 0.001** |  | **0.563 (0.490,0.648)** | **< 0.001** |  | **0.660 (0.565,0.772)** | **< 0.001** |  | 691/9,404 | **0.563 (0.498,0.636)** | **< 0.001** |  | **0.565 (0.497,0.642)** | **< 0.001** |  | **0.726 (0.629,0.838)** | **< 0.001** |
| *P* for trend | - | - | **< 0.001** |  | - | **< 0.001** |  | - | **< 0.001** |  | - | - | **< 0.001** |  | - | **< 0.001** |  | - | **< 0.001** |
| **Nicotine exposure** |  |  |  |  |  |  |  |  |  |  |  |  |  |  |  |  |  |  |  |
| Unfavorable score | 405/2,737 | Reference | - |  | Reference | - |  | Reference | - |  | 493/3,042 | Reference | - |  | Reference | - |  | Reference | - |
| Intermediate score | 178/2,757 | **0.397 (0.330,0.477)** | **< 0.001** |  | **0.467 (0.384,0.566)** | **< 0.001** |  | **0.661 (0.529,0.824)** | **< 0.001** |  | 245/2,950 | **0.468 (0.398,0.550)** | **< 0.001** |  | **0.464 (0.391,0.549)** | **< 0.001** |  | **0.636 (0.525,0.768)** | **< 0.001** |
| Favorable score | 389/6,008 | **0.399 (0.344,0.462)** | **< 0.001** |  | **0.370 (0.318,0.431)** | **< 0.001** |  | **0.575 (0.478,0.691)** | **< 0.001** |  | 484/7,863 | **0.339 (0.297,0.387)** | **< 0.001** |  | **0.298 (0.259,0.342)** | **< 0.001** |  | **0.469 (0.399,0.551)** | **< 0.001** |
| *P* for trend | - | - | **< 0.001** |  | - | **< 0.001** |  | - | **< 0.001** |  | - | - | **< 0.001** |  | - | **< 0.001** |  | - | **< 0.001** |
| **Sleep health** |  |  |  |  |  |  |  |  |  |  |  |  |  |  |  |  |  |  |  |
| Unfavorable score | 375/2,059 | Reference | - |  | Reference | - |  | Reference | - |  | 421/2,408 | Reference | - |  | Reference | - |  | Reference | - |
| Intermediate score | 229/2,709 | **0.415 (0.348,0.494)** | **< 0.001** |  | **0.405 (0.339,0.483)** | **< 0.001** |  | **0.495 (0.406,0.602)** | **< 0.001** |  | 235/2,747 | **0.442 (0.372,0.523)** | **< 0.001** |  | **0.433 (0.365,0.514)** | **< 0.001** |  | **0.514 (0.425,0.620)** | **< 0.001** |
| Favorable score | 368/6,734 | **0.260 (0.223,0.303)** | **< 0.001** |  | **0.255 (0.217,0.298)** | **< 0.001** |  | **0.338 (0.285,0.402)** | **< 0.001** |  | 566/8,700 | **0.328 (0.287,0.376)** | **< 0.001** |  | **0.315 (0.274,0.361)** | **< 0.001** |  | **0.417 (0.359,0.485)** | **< 0.001** |
| *P* for trend | - | - | **< 0.001** |  | - | **< 0.001** |  | - | **< 0.001** |  | - | - | **< 0.001** |  | - | **< 0.001** |  | - | **< 0.001** |
| **BMI** |  |  |  |  |  |  |  |  |  |  |  |  |  |  |  |  |  |  |  |
| Unfavorable score | 459/4,327 | Reference | - |  | Reference | - |  | Reference | - |  | 661/5,608 | Reference | - |  | Reference | - |  | Reference | - |
| Intermediate score | 275/3,945 | **0.631 (0.540,0.738)** | **< 0.001** |  | **0.691 (0.590,0.810)** | **< 0.001** |  | **0.793 (0.662,0.949)** | **< 0.05** |  | 291/4,396 | **0.531 (0.459,0.612)** | **< 0.001** |  | **0.564 (0.487,0.652)** | **< 0.001** |  | **0.659 (0.560,0.775)** | **< 0.001** |
| Favorable score | 238/3,230 | **0.670 (0.568,0.789)** | **< 0.001** |  | **0.686 (0.579,0.810)** | **< 0.001** |  | **0.820 (0.674,0.995)** | **< 0.05** |  | 270/3,851 | **0.564 (0.486,0.653)** | **< 0.001** |  | **0.568 (0.487,0.660)** | **< 0.001** |  | **0.663 (0.556,0.790)** | **< 0.001** |
| *P* for trend | - | - | **< 0.001** |  | - | **< 0.001** |  | - | **< 0.05** |  | - | - | **< 0.001** |  | - | **< 0.001** |  | - | **< 0.001** |
| **Blood lipids (non–HDL-C)** |  |  |  |  |  |  |  |  |  |  |  |  |  |  |  |  |  |  |  |
| Unfavorable score | 432/4,543 | Reference | - |  | Reference | - |  | Reference | - |  | 486/4,787 | Reference | - |  | Reference | - |  | Reference | - |
| Intermediate score | 190/2,586 | **0.755 (0.630,0.900)** | **< 0.05** |  | **0.703 (0.586,0.841)** | **< 0.001** |  | **0.816 (0.667,0.995)** | **< 0.05** |  | 256/3,027 | **0.818 (0.697,0.957)** | **< 0.05** |  | **0.808 (0.687,0.948)** | **< 0.05** |  | 0.932 (0.780,1.112) | 0.439 |
| Favorable score | 350/4,373 | **0.828 (0.714,0.959)** | **< 0.05** |  | **0.822 (0.706,0.957)** | **< 0.05** |  | 0.971 (0.818,1.153) | 0.739 |  | 480/6,041 | **0.764 (0.669,0.872)** | **< 0.001** |  | **0.772 (0.674,0.884)** | **< 0.001** |  | 0.877 (0.753,1.020) | 0.089 |
| *P* for trend | - | - | **< 0.05** |  | - | **< 0.05** |  | - | 0.719 |  | - | - | **< 0.001** |  | - | **< 0.001** |  | - | 0.089 |
| **Blood glucose** |  |  |  |  |  |  |  |  |  |  |  |  |  |  |  |  |  |  |  |
| Unfavorable score | 239/2,011 | Reference | - |  | Reference | - |  | Reference | - |  | 326/2,660 | Reference | - |  | Reference | - |  | Reference | - |
| Intermediate score | 170/2,138 | **0.640 (0.520,0.787)** | **< 0.001** |  | **0.632 (0.512,0.779)** | **< 0.001** |  | **0.667 (0.527,0.844)** | **< 0.001** |  | 234/2,881 | **0.633 (0.530,0.755)** | **< 0.001** |  | **0.615 (0.515,0.735)** | **< 0.001** |  | **0.617 (0.506,0.752)** | **< 0.001** |
| Favorable score | 563/7,353 | **0.615 (0.524,0.723)** | **< 0.001** |  | **0.512 (0.431,0.609)** | **< 0.001** |  | **0.641 (0.521,0.791)** | **< 0.001** |  | 662/8,314 | **0.619 (0.539,0.713)** | **< 0.001** |  | **0.556 (0.478,0.648)** | **< 0.001** |  | **0.711 (0.594,0.853)** | **< 0.001** |
| *P* for trend | - | - | **< 0.001** |  | - | **< 0.001** |  | - | **< 0.001** |  | - | - | **< 0.001** |  | - | **< 0.001** |  | - | **< 0.05** |
| **Blood pressure** |  |  |  |  |  |  |  |  |  |  |  |  |  |  |  |  |  |  |  |
| Unfavorable score | 240/2,801 | Reference | - |  | Reference | - |  | Reference | - |  | 347/3,506 | Reference | - |  | Reference | - |  | Reference | - |
| Intermediate score | 265/3,632 | 0.840 (0.700,1.008) | 0.060 |  | **0.731 (0.604,0.884)** | **< 0.001** |  | 0.845 (0.683,1.045) | 0.119 |  | 362/4,511 | **0.794 (0.681,0.927)** | **< 0.05** |  | **0.773 (0.658,0.908)** | **< 0.05** |  | 0.883 (0.739,1.055) | 0.170 |
| Favorable score | 467/5,069 | 1.083 (0.921,1.276) | 0.338 |  | 0.844 (0.705,1.012) | 0.066 |  | 1.032 (0.843,1.265) | 0.760 |  | 513/5,838 | 0.877 (0.760,1.013) | 0.072 |  | **0.788 (0.672,0.924)** | **< 0.05** |  | 1.024 (0.859,1.222) | 0.792 |
| *P* for trend | - | - | 0.140 |  | - | 0.207 |  | - | 0.451 |  | - | - | 0.139 |  | - | **< 0.05** |  | - | 0.609 |
| Footnotes: The total LE8 score was calculated as the mean of the sum of all 8 items of LE8 score and similarly ranged from 0 (if the mean score of all items was 0) to 100 (optimal CVH). Score for total LE8 score was categorized into unfavorable score (0-49 points), intermediate score (50-79 points), and favorable score (80-100 points) according to AHA recommendation^[1]^. For each sub-item of LE8 score: Crude model was unadjusted. Model 1 was adjusted for age, gender, and race/ethnicity. Model 2 was adjusted for age, gender, race/ethnicity, PIR, education level, alcohol consumption, marital status. In the case of total LE8 score, Model 2 was adjusted for age, gender, race/ethnicity, PIR, education level, alcohol consumption, and marital status. Results of COR (95% CI), AOR (95% CI), *P* for tend, and *P*-value presented with bold valued were statistically significant with *P*-value < 0.05 or *P*-value < 0.001. ^[1]^ Lloyd-Jones DM, Allen NB, Anderson CAM, et al. Life's Essential 8: Updating and Enhancing the American Heart Association's Construct of Cardiovascular Health: A Presidential Advisory From the American Heart Association. Circulation. 2022;146(5):e18-e43. doi:10.1161/CIR.0000000000001078 | | | | | | | | | | | | | | | | | | | |
| Abbreviations: AHA, American Heart Association; AOR, Adjusted odds ratio; BMI, Body mass index; CI, Confidence interval; COR, Crude odds ratio; CVH, Cardiovascular health; LE8, Life’s Essential 8; NHANES, National Health and Nutrition Examination Survey; Non-HDL-C, Non-high-density lipoprotein cholesterol; PA, Physical activity; PIR, Poverty-to-income ratio. | | | | | | | | | | | | | | | | | | | |

| **Supplementary Table S10** Further adjustments for total and individual LE8 score with risk of depressive symptom in survey-weighted logistic regression models, NHANES 2005-2018, U.S (n = 25,357) | | | | | | | | | | | | | | | | | | | | |
| --- | --- | --- | --- | --- | --- | --- | --- | --- | --- | --- | --- | --- | --- | --- | --- | --- | --- | --- | --- | --- |
| **LE8 variables** | **Adjusted for survey waves** | |  | **Adjusted for DM** | |  | **Adjusted for hypertension** | |  | **Adjusted for CVDs** | |  | **Adjusted for**  **DM + hypertension + CVDs** | |  | **Adjusted for**  **whether using antidepressant** | |  | **Adjusted for**  **total energy intake** | |
|  | **AOR (95% CI)** | ***P*-value** |  | **AOR (95% CI)** | ***P*-value** |  | **AOR (95% CI)** | ***P*-value** |  | **AOR (95% CI)** | ***P*-value** |  | **AOR (95% CI)** | ***P*-value** |  | **AOR (95% CI)** | ***P*-value** |  | **AOR (95% CI)** | ***P*-value** |
| **Total** |  |  |  |  |  |  |  |  |  |  |  |  |  |  |  |  |  |  |  |  |
| Unfavorable score | Reference | - |  | Reference | - |  | Reference | - |  | Reference | - |  | Reference | - |  | Reference | - |  | Reference | - |
| Intermediate score | **0.459 (0.413,0.509)** | **< 0.001** |  | **0.474 (0.426,0.528)** | **< 0.001** |  | **0.494 (0.444,0.550)** | **< 0.001** |  | **0.510 (0.458,0.568)** | **< 0.001** |  | **0.518 (0.464,0.579)** | **< 0.001** |  | **0.505 (0.454,0.563)** | **< 0.001** |  | **0.482 (0.433,0.537)** | **< 0.001** |
| Favorable score | **0.212 (0.175,0.256)** | **< 0.001** |  | **0.227 (0.187,0.275)** | **< 0.001** |  | **0.236 (0.194,0.285)** | **< 0.001** |  | **0.251 (0.206,0.305)** | **< 0.001** |  | **0.257 (0.210,0.313)** | **< 0.001** |  | **0.260 (0.213,0.315)** | **< 0.001** |  | **0.237 (0.195,0.287)** | **< 0.001** |
| *P* for trend | - | **< 0.001** |  | - | **< 0.001** |  | - | **< 0.001** |  | - | **< 0.001** |  | - | **< 0.001** |  | - | **< 0.001** |  | - | **< 0.001** |
| **Diet** |  |  |  |  |  |  |  |  |  |  |  |  |  |  |  |  |  |  |  |  |
| Unfavorable score | Reference | - |  | Reference | - |  | Reference | - |  | Reference | - |  | Reference | - |  | Reference | - |  | Reference | - |
| Intermediate score | 0.889 (0.787,1.004) | 0.059 |  | 0.890 (0.787,1.004) | 0.060 |  | 0.892 (0.789,1.007) | 0.065 |  | 0.896 (0.792,1.011) | 0.076 |  | 0.895 (0.791,1.010) | 0.074 |  | 0.894 (0.791,1.010) | 0.074 |  | 0.890 (0.788,1.004) | 0.060 |
| Favorable score | **0.752 (0.653,0.865)** | **< 0.001** |  | **0.755 (0.655,0.869)** | **< 0.001** |  | **0.755 (0.655,0.868)** | **< 0.001** |  | **0.766 (0.664,0.881)** | **< 0.001** |  | **0.763 (0.662,0.878)** | **< 0.001** |  | **0.751 (0.652,0.864)** | **< 0.001** |  | **0.753 (0.653,0.866)** | **< 0.001** |
| *P* for trend | - | **< 0.001** |  | - | **< 0.001** |  | - | **< 0.001** |  | - | **< 0.001** |  | - | **< 0.001** |  | - | **< 0.001** |  | - | **< 0.001** |
| **PA** |  |  |  |  |  |  |  |  |  |  |  |  |  |  |  |  |  |  |  |  |
| Unfavorable score | Reference | - |  | Reference | - |  | Reference | - |  | Reference | - |  | Reference | - |  | Reference | - |  | Reference | - |
| Intermediate score | **0.702 (0.543,0.896)** | **< 0.05** |  | **0.683 (0.529,0.871)** | **< 0.05** |  | **0.687 (0.532,0.877)** | **< 0.05** |  | **0.708 (0.547,0.904)** | **< 0.05** |  | **0.713 (0.551,0.910)** | **< 0.05** |  | **0.678 (0.524,0.866)** | **< 0.05** |  | **0.682 (0.528,0.870)** | **< 0.05** |
| Favorable score | **0.671 (0.603,0.746)** | **< 0.001** |  | **0.702 (0.632,0.780)** | **< 0.001** |  | **0.706 (0.635,0.784)** | **< 0.001** |  | **0.723 (0.651,0.804)** | **< 0.001** |  | **0.727 (0.654,0.809)** | **< 0.001** |  | **0.715 (0.643,0.794)** | **< 0.001** |  | **0.702 (0.632,0.780)** | **< 0.001** |
| *P* for trend | - | **< 0.001** |  | - | **< 0.001** |  | - | **< 0.001** |  | - | **< 0.001** |  | - | **< 0.001** |  | - | **< 0.001** |  | - | **< 0.001** |
| **Nicotine exposure** |  |  |  |  |  |  |  |  |  |  |  |  |  |  |  |  |  |  |  |  |
| Unfavorable score | Reference | - |  | Reference | - |  | Reference | - |  | Reference | - |  | Reference | - |  | Reference | - |  | Reference | - |
| Intermediate score | **0.646 (0.559,0.745)** | **< 0.001** |  | **0.644 (0.557,0.743)** | **< 0.001** |  | **0.645 (0.558,0.744)** | **< 0.001** |  | **0.644 (0.557,0.744)** | **< 0.001** |  | **0.644 (0.557,0.744)** | **< 0.001** |  | **0.630 (0.545,0.727)** | **< 0.001** |  | **0.644 (0.557,0.743)** | **< 0.001** |
| Favorable score | **0.507 (0.449,0.572)** | **< 0.001** |  | **0.509 (0.450,0.574)** | **< 0.001** |  | **0.513 (0.454,0.579)** | **< 0.001** |  | **0.521 (0.461,0.588)** | **< 0.001** |  | **0.524 (0.464,0.592)** | **< 0.001** |  | **0.521 (0.461,0.588)** | **< 0.001** |  | **0.508 (0.450,0.573)** | **< 0.001** |
| *P* for trend | - | **< 0.001** |  | - | **< 0.001** |  | - | **< 0.001** |  | - | **< 0.001** |  | - | **< 0.001** |  | - | **< 0.001** |  | - | **< 0.001** |
| **Sleep health** |  |  |  |  |  |  |  |  |  |  |  |  |  |  |  |  |  |  |  |  |
| Unfavorable score | Reference | - |  | Reference | - |  | Reference | - |  | Reference | - |  | Reference | - |  | Reference | - |  | Reference | - |
| Intermediate score | **0.506 (0.442,0.580)** | **< 0.001** |  | **0.507 (0.443,0.581)** | **< 0.001** |  | **0.511 (0.446,0.585)** | **< 0.001** |  | **0.516 (0.450,0.591)** | **< 0.001** |  | **0.520 (0.453,0.596)** | **< 0.001** |  | **0.517 (0.451,0.592)** | **< 0.001** |  | **0.507 (0.442,0.580)** | **< 0.001** |
| Favorable score | **0.384 (0.343,0.430)** | **< 0.001** |  | **0.385 (0.344,0.431)** | **< 0.001** |  | **0.390 (0.349,0.437)** | **< 0.001** |  | **0.394 (0.352,0.441)** | **< 0.001** |  | **0.399 (0.356,0.447)** | **< 0.001** |  | **0.395 (0.352,0.442)** | **< 0.001** |  | **0.385 (0.344,0.431)** | **< 0.001** |
| *P* for trend | - | **< 0.001** |  | - | **< 0.001** |  | - | **< 0.001** |  | - | **< 0.001** |  | - | **< 0.001** |  | - | **< 0.001** |  | - | **< 0.001** |
| **BMI** |  |  |  |  |  |  |  |  |  |  |  |  |  |  |  |  |  |  |  |  |
| Unfavorable score | Reference | - |  | Reference | - |  | Reference | - |  | Reference | - |  | Reference | - |  | Reference | - |  | Reference | - |
| Intermediate score | **0.716 (0.634,0.807)** | **< 0.001** |  | **0.715 (0.633,0.806)** | **< 0.001** |  | **0.729 (0.646,0.822)** | **< 0.001** |  | **0.722 (0.640,0.815)** | **< 0.001** |  | **0.738 (0.654,0.833)** | **< 0.001** |  | **0.736 (0.652,0.831)** | **< 0.001** |  | **0.710 (0.630,0.801)** | **< 0.001** |
| Favorable score | **0.731 (0.641,0.832)** | **< 0.001** |  | **0.726 (0.637,0.826)** | **< 0.001** |  | **0.751 (0.659,0.856)** | **< 0.001** |  | **0.736 (0.646,0.838)** | **< 0.001** |  | **0.762 (0.668,0.868)** | **< 0.001** |  | **0.753 (0.660,0.857)** | **< 0.001** |  | **0.722 (0.634,0.821)** | **< 0.001** |
| *P* for trend | - | **< 0.001** |  | - | **< 0.001** |  | - | **< 0.001** |  | - | **< 0.001** |  | - | **< 0.001** |  | - | 0.159 |  | - | **< 0.001** |
| **Blood lipids (non–HDL-C)** |  |  |  |  |  |  |  |  |  |  |  |  |  |  |  |  |  |  |  |  |
| Unfavorable score | Reference | - |  | Reference | - |  | Reference | - |  | Reference | - |  | Reference | - |  | Reference | - |  | Reference | - |
| Intermediate score | 0.884 (0.774,1.009) | 0.069 |  | 0.887 (0.777,1.012) | 0.075 |  | 0.895 (0.784,1.021) | 0.101 |  | 0.900 (0.788,1.028) | 0.121 |  | 0.905 (0.792,1.033) | 0.142 |  | 0.911 (0.797,1.041) | 0.172 |  | 0.889 (0.778,1.014) | 0.080 |
| Favorable score | 0.917 (0.819,1.028) | 0.136 |  | 0.922 (0.824,1.033) | 0.161 |  | 0.918 (0.820,1.028) | 0.138 |  | **0.892 (0.796,0.999)** | **< 0.05** |  | **0.886 (0.790,0.993)** | **< 0.05** |  | 0.903 (0.806,1.012) | 0.079 |  | 0.926 (0.827,1.037) | 0.183 |
| *P* for trend | - | 0.143 |  | - | 0.169 |  | - | 0.144 |  | - | 0.051 |  | - | **< 0.05** |  | - | 0.082 |  | - | 0.192 |
| **Blood glucose** |  |  |  |  |  |  |  |  |  |  |  |  |  |  |  |  |  |  |  |  |
| Unfavorable score | Reference | - |  | Reference | - |  | Reference | - |  | Reference | - |  | Reference | - |  | Reference | - |  | Reference | - |
| Intermediate score | **0.634 (0.545,0.738)** | **< 0.001** |  | **0.612 (0.507,0.736)** | **< 0.001** |  | **0.655 (0.563,0.762)** | **< 0.001** |  | **0.681 (0.585,0.794)** | **< 0.001** |  | **0.658 (0.544,0.794)** | **< 0.001** |  | **0.714 (0.613,0.832)** | **< 0.001** |  | **0.637 (0.548,0.741)** | **< 0.001** |
| Favorable score | **0.686 (0.598,0.787)** | **< 0.001** |  | **0.642 (0.532,0.773)** | **< 0.001** |  | **0.695 (0.606,0.797)** | **< 0.001** |  | **0.727 (0.633,0.835)** | **< 0.001** |  | **0.697 (0.576,0.841)** | **< 0.001** |  | **0.769 (0.671,0.883)** | **< 0.001** |  | **0.673 (0.587,0.772)** | **< 0.001** |
| *P* for trend | - | **< 0.001** |  | - | **< 0.001** |  | - | **< 0.001** |  | - | **< 0.001** |  | - | **< 0.05** |  | - | **< 0.001** |  | - | **< 0.001** |
| **Blood pressure** |  |  |  |  |  |  |  |  |  |  |  |  |  |  |  |  |  |  |  |  |
| Unfavorable score | Reference | - |  | Reference | - |  | Reference | - |  | Reference | - |  | Reference | - |  | Reference | - |  | Reference | - |
| Intermediate score | **0.863 (0.753,0.988)** | **< 0.05** |  | **0.871 (0.761,0.998)** | **< 0.05** |  | 0.986 (0.853,1.139) | 0.847 |  | 0.910 (0.794,1.044) | 0.180 |  | 1.008 (0.872,1.166) | 0.911 |  | 0.981 (0.855,1.125) | 0.785 |  | **0.867 (0.757,0.993)** | **< 0.05** |
| Favorable score | 1.019 (0.892,1.163) | 0.784 |  | 1.028 (0.900,1.174) | 0.686 |  | **1.202 (1.038,1.391)** | **< 0.05** |  | 1.046 (0.916,1.196) | 0.505 |  | **1.188 (1.026,1.376)** | **< 0.05** |  | 1.117 (0.979,1.276) | 0.102 |  | 1.022 (0.895,1.167) | 0.750 |
| *P* for trend | - | 0.463 |  | - | 0.390 |  | - | **< 0.05** |  | - | 0.304 |  | - | **< 0.05** |  | - | 0.061 |  |  | 0.439 |
| Footnotes: The total LE8 score was calculated as the mean of the sum of all 8 items of LE8 score and similarly ranged from 0 (if the mean score of all items was 0) to 100 (optimal CVH). Score for total LE8 score was categorized into unfavorable score (0-49 points), intermediate score (50-79 points), and favorable score (80-100 points) according to AHA recommendation^[1]^. For each sub-item of LE8 score: Model 2 was adjusted for age, gender, race/ethnicity, PIR, education level, alcohol consumption, marital status. In the case of total LE8 score, Model 2 was adjusted for age, gender, race/ethnicity, PIR, education level, alcohol consumption, and marital status. Sensitivity analyses were performed based on Model 2, and plus adjusted by adding variables including survey waves, DM, hypertension, CVDs, DM + hypertension + CVDs, whether using antidepressant, and total energy intake, respectively. Results of AOR (95% CI), *P* for trend, and *P*-value presented with bold valued were statistically significant with *P*-value < 0.05 or *P*-value < 0.001.  ^[1]^ Lloyd-Jones DM, Allen NB, Anderson CAM, et al. Life's Essential 8: Updating and Enhancing the American Heart Association's Construct of Cardiovascular Health: A Presidential Advisory From the American Heart Association. Circulation. 2022;146(5):e18-e43. doi:10.1161/CIR.0000000000001078 | | | | | | | | | | | | | | | | | | | | |
| Abbreviations: AHA, American Heart Association; AOR, Adjusted odds ratio; BMI, Body mass index; CI, Confidence interval; CVDs, Cardiovascular diseases; CVH, Cardiovascular health; DM, Diabetes mellitus; LE8, Life’s Essential 8; NHANES, National Health and Nutrition Examination Survey; Non-HDL-C, Non-high-density lipoprotein cholesterol; PA, Physical activity; PIR, Poverty-to-income ratio. | | | | | | | | | | | | | | | | | | | | |

| **Supplementary Table S11** Associations between total and individual LE8 score with risk of depressive symptom grouped by median values of score, NHANES 2005-2018, U.S (n = 25,357) | | | | | | | | | |
| --- | --- | --- | --- | --- | --- | --- | --- | --- | --- |
| **LE8 variables** | **Cases/participants** | **Crude model** | |  | **Model 1** | |  | **Model 2** | |
|  |  | **COR (95% CI)** | ***P*-value** |  | **AOR (95% CI)** | ***P*-value** |  | **AOR (95% CI)** | ***P*-value** |
| **Total** |  |  |  |  |  |  |  |  |  |
| < median value of score | 1,587/12,851 | Reference | - |  | Reference | - |  | Reference | - |
| > median value of score | 607/12,506 | **0.362 (0.328,0.399)** | **< 0.001** |  | **0.322 (0.291,0.356)** | **< 0.001** |  | **0.415 (0.374,0.460)** | **< 0.001** |
| **Diet** |  |  |  |  |  |  |  |  |  |
| < median value of score | 1,360/12,782 | Reference | - |  | Reference | - |  | Reference | - |
| > median value of score | 834/12,575 | **0.597 (0.545,0.653)** | **< 0.001** |  | **0.574 (0.523,0.630)** | **< 0.001** |  | **0.833 (0.750,0.924)** | **< 0.001** |
| **PA** |  |  |  |  |  |  |  |  |  |
| < median value of score | 947/8,083 | Reference | - |  | Reference | - |  | Reference | - |
| > median value of score | 1,247/17,274 | **0.586 (0.536,0.641)** | **< 0.001** |  | **0.570 (0.520,0.625)** | **< 0.001** |  | **0.699 (0.630,0.775)** | **< 0.001** |
| **Nicotine exposure** |  |  |  |  |  |  |  |  |  |
| < median value of score | 977/6,542 | Reference | - |  | Reference | - |  | Reference | - |
| > median value of score | 1,217/18,807 | **0.394 (0.360,0.431)** | **< 0.001** |  | **0.369 (0.336,0.405)** | **< 0.001** |  | **0.544 (0.488,0.607)** | **< 0.001** |
| **Sleep health** |  |  |  |  |  |  |  |  |  |
| < median value of score | 342/1,264 | Reference | - |  | Reference | - |  | Reference | - |
| > median value of score | 1,834/24,035 | **0.223 (0.195,0.255)** | **< 0.001** |  | **0.224 (0.195,0.256)** | **< 0.001** |  | **0.304 (0.262,0.354)** | **< 0.001** |
| **BMI** |  |  |  |  |  |  |  |  |  |
| < median value of score | 1,686/18,276 | Reference | - |  | Reference | - |  | Reference | - |
| > median value of score | 508/7,081 | **0.760 (0.685,0.843)** | **< 0.001** |  | **0.743 (0.668,0.825)** | **< 0.001** |  | **0.839 (0.743,0.944)** | **< 0.05** |
| **Blood lipids (non–HDL-C)** | |  |  |  |  |  |  |  |  |
| < median value of score | 826/8,331 | Reference | - |  | Reference | - |  | Reference | - |
| > median value of score | 1,276/16,027 | **0.786 (0.717,0.862)** | **< 0.001** |  | **0.770 (0.701,0.847)** | **< 0.001** |  | 0.917 (0.826,1.017) | 0.100 |
| **Blood glucose** |  |  |  |  |  |  |  |  |  |
| < median value of score | 417/3,139 | Reference | - |  | Reference | - |  | Reference | - |
| > median value of score | 1,629/20,686 | **0.558 (0.498,0.626)** | **< 0.001** |  | **0.484 (0.428,0.547)** | **< 0.001** |  | **0.626 (0.546,0.718)** | **< 0.001** |
| **Blood pressure** |  |  |  |  |  |  |  |  |  |
| < median value of score | 830/9,793 | Reference | - |  | Reference | - |  | Reference | - |
| > median value of score | 1,314/15,146 | 1.026 (0.937,1.124) | 0.582 |  | 0.934 (0.848,1.029) | 0.167 |  | 1.057 (0.951,1.176) | 0.306 |

Footnotes: The total LE8 score was calculated as the mean of the sum of all 8 items of LE8 score and similarly ranged from 0 (if the mean score of all items was 0) to 100 (optimal CVH). Score for total LE8 and its items was categorized into two groups (< median value of score, > median value of score) based on median value of score for sensitivity analyses. For each sub-item of LE8 score: Crude model was unadjusted. Model 1 was adjusted for age, gender, and race/ethnicity. Model 2 was adjusted for age, gender, race/ethnicity, PIR, education level, alcohol consumption, marital status. In the case of total LE8 score, Model 2 was adjusted for age, gender, race/ethnicity, PIR, education level, alcohol consumption, and marital status. Results of COR (95% CI), AOR (95% CI), and *P*-value presented with bold valued were statistically significant with *P*-value < 0.05 or *P*-value < 0.001.

Abbreviations: AOR, Adjusted odds ratio; BMI, Body mass index; CI, Confidence interval; COR, Crude odds ratio; CVH, Cardiovascular health; LE8, Life’s Essential 8; NHANES, National Health and Nutrition Examination Survey; Non-HDL-C, Non-high-density lipoprotein cholesterol; PA, Physical activity; PIR, Poverty-to-income ratio.

| **Supplementary Table S12** Sensitivity analyses for total and individual LE8 score associated with risk of depressive symptom by using favorable score as the reference, NHANES 2005-2018, U.S (n = 25,357) | | | | | | | | | |
| --- | --- | --- | --- | --- | --- | --- | --- | --- | --- |
| **LE8 variables** | **Cases/participants** | **Crude model** | |  | **Model 1** | |  | **Model 2** | |
|  |  | **COR (95% CI)** | ***P*-value** |  | **AOR (95% CI)** | ***P*-value** |  | **AOR (95% CI)** | ***P*-value** |
| **Total** |  |  |  |  |  |  |  |  |  |
| Favorable score | 163/4,549 | Reference | - |  | Reference | - |  | Reference | - |
| Intermediate score | 1,314/16,765 | **2.288 (1.944,2.711)** | **< 0.001** |  | **2.739 (2.321,3.254)** | **< 0.001** |  | **2.125 (1.793,2.535)** | **< 0.001** |
| Unfavorable score | 717/4,043 | **5.801 (4.878,6.937)** | **< 0.001** |  | **7.355 (6.143,8.854)** | **< 0.001** |  | **4.639 (3.846,5.624)** | **< 0.001** |
| *P* for trend | - | - | **< 0.001** |  | - | **< 0.001** |  | - | **< 0.001** |
| **Diet** |  |  |  |  |  |  |  |  |  |
| Favorable score | 351/6,273 | Reference | - |  | Reference | - |  | Reference | - |
| Intermediate score | 483/6,302 | **1.400 (1.215,1.615)** | **< 0.001** |  | **1.449 (1.256,1.673)** | **< 0.001** |  | **1.178 (1.007,1.380)** | **< 0.05** |
| Unfavorable score | 1,360/12,782 | **2.009 (1.781,2.271)** | **< 0.001** |  | **2.131 (1.884,2.416)** | **< 0.001** |  | **1.322 (1.150,1.524)** | **< 0.001** |
| *P* for trend | - | - | **< 0.001** |  | - | **< 0.001** |  | - | **< 0.001** |
| **PA** |  |  |  |  |  |  |  |  |  |
| Favorable score | 1,151/16,068 | Reference | - |  | Reference | - |  | Reference | - |
| Intermediate score | 96/1,206 | 1.121 (0.897,1.385) | 0.303 |  | 1.076 (0.860,1.331) | 0.511 |  | 0.973 (0.756,1.237) | 0.826 |
| Unfavorable score | 947/8,083 | **1.720 (1.571,1.883)** | **< 0.001** |  | **1.765 (1.608,1.938)** | **< 0.001** |  | **1.428 (1.285,1.585)** | **< 0.001** |
| *P* for trend | - | - | **< 0.001** |  | - | **< 0.001** |  | - | **< 0.001** |
| **Nicotine exposure** |  |  |  |  |  |  |  |  |  |
| Favorable score | 873/13,871 | Reference | - |  | Reference | - |  | Reference | - |
| Intermediate score | 423/5,707 | **1.192 (1.056,1.344)** | **< 0.05** |  | **1.415 (1.247,1.604)** | **< 0.001** |  | **1.268 (1.102,1.457)** | **< 0.001** |
| Unfavorable score | 898/5,779 | **2.739 (2.482,3.024)** | **< 0.001** |  | **3.055 (2.757,3.385)** | **< 0.001** |  | **1.966 (1.742,2.219)** | **< 0.001** |
| *P* for trend | - | - | **< 0.001** |  | - | **< 0.001** |  | - | **< 0.001** |
| **Sleep health** |  |  |  |  |  |  |  |  |  |
| Favorable score | 934/15,434 | Reference | - |  | Reference | - |  | Reference | - |
| Intermediate score | 464/5,456 | **1.443 (1.284,1.619)** | **< 0.001** |  | **1.459 (1.297,1.639)** | **< 0.001** |  | **1.317 (1.159,1.494)** | **< 0.001** |
| Unfavorable score | 796/4,467 | **3.366 (3.042,3.725)** | **< 0.001** |  | **3.481 (3.139,3.859)** | **< 0.001** |  | **2.602 (2.324,2.912)** | **< 0.001** |
| *P* for trend | - | - | **< 0.001** |  | - | **< 0.001** |  | - | **< 0.001** |
| **BMI** |  |  |  |  |  |  |  |  |  |
| Favorable score | 508/7,081 | Reference | - |  | Reference | - |  | Reference | - |
| Intermediate score | 566/8,341 | 0.942 (0.832,1.067) | 0.345 |  | 1.007 (0.887,1.142) | 0.919 |  | 0.985 (0.857,1.132) | 0.835 |
| Unfavorable score | 1,120/9,935 | **1.644 (1.474,1.836)** | **< 0.001** |  | **1.630 (1.458,1.825)** | **< 0.001** |  | **1.386 (1.218,1.578)** | **< 0.001** |
| *P* for trend | - | - | **< 0.001** |  | - | **< 0.001** |  | - | **< 0.001** |
| **Blood lipids (non–HDL-C)** | |  |  |  |  |  |  |  |  |
| Favorable score | 830/10,414 | Reference | - |  | Reference | - |  | Reference | - |
| Intermediate score | 446/5,613 | 0.997 (0.883,1.123) | 0.957 |  | 0.956 (0.847,1.079) | 0.471 |  | 0.959 (0.841,1.092) | 0.531 |
| Unfavorable score | 918/9,330 | **1.260 (1.142,1.390)** | **< 0.001** |  | **1.258 (1.137,1.391)** | **< 0.001** |  | 1.081 (0.965,1.210) | 0.178 |
| *P* for trend | - | - | **< 0.001** |  | - | **< 0.001** |  | - | 0.187 |
| **Blood glucose** |  |  |  |  |  |  |  |  |  |
| Favorable score | 1,225/15,667 | Reference | - |  | Reference | - |  | Reference | - |
| Intermediate score | 404/5,019 | 1.032 (0.917,1.159) | 0.598 |  | **1.157 (1.022,1.308)** | **< 0.05** |  | 0.947 (0.828,1.081) | 0.421 |
| Unfavorable score | 565/4,671 | **1.622 (1.459,1.802)** | **< 0.001** |  | **1.871 (1.669,2.095)** | **< 0.001** |  | **1.488 (1.298,1.705)** | **< 0.001** |
| *P* for trend | - | - | **< 0.001** |  | - | **< 0.001** |  | - | **< 0.001** |
| **Blood pressure** |  |  |  |  |  |  |  |  |  |
| Favorable score | 980/10,907 | Reference | - |  | Reference | - |  | Reference | - |
| Intermediate score | 627/8,143 | **0.845 (0.761,0.938)** | **< 0.05** |  | 0.928 (0.833,1.034) | 0.175 |  | **0.846 (0.752,0.952)** | **< 0.05** |
| Unfavorable score | 587/6,307 | 1.040 (0.933,1.157) | 0.479 |  | **1.225 (1.087,1.380)** | **< 0.001** |  | 0.978 (0.856,1.116) | 0.741 |
| *P* for trend | - | - | 0.889 |  | - | **< 0.05** |  | - | 0.428 |
| Footnotes: The total LE8 score was calculated as the mean of the sum of all 8 items of LE8 score and similarly ranged from 0 (if the mean score of all items was 0) to 100 (optimal CVH). Score for total LE8 score and its items was categorized into unfavorable score (0-49 points), intermediate score (50-79 points), and favorable score (80-100 points) according to AHA recommendation^[1]^. For each sub-item of LE8 score: Crude model was unadjusted. Model 1 was adjusted for age, gender, and race/ethnicity. Model 2 was adjusted for age, gender, race/ethnicity, PIR, education level, alcohol consumption, marital status. In the case of total LE8 score, Model 2 was adjusted for age, gender, race/ethnicity, PIR, education level, alcohol consumption, and marital status. Results of COR (95% CI), AOR (95% CI), *P* for trend, and *P*-value presented with bold valued were statistically significant with *P*-value < 0.05 or *P*-value < 0.001. ^[1]^ Lloyd-Jones DM, Allen NB, Anderson CAM, et al. Life's Essential 8: Updating and Enhancing the American Heart Association's Construct of Cardiovascular Health: A Presidential Advisory From the American Heart Association. Circulation. 2022;146(5): e18-e43. doi:10.1161/CIR.0000000000001078 | | | | | | | | | |
| Abbreviations: AHA, American Heart Association; AOR, Adjusted odds ratio; BMI, Body mass index; CI, Confidence interval; COR, Crude odds ratio; CVH, Cardiovascular health; LE8, Life’s Essential 8; NHANES, National Health and Nutrition Examination Survey; Non-HDL-C, Non-high-density lipoprotein cholesterol; PA, Physical activity; PIR, Poverty-to-income ratio. | | | | | | | | | |

| **Supplementary Table S13** Sensitivity analyses for total and individual LE8 score (continuous variable) associated with risk of depressive symptom, NHANES 2005-2018, U.S (n = 25,357) | | |
| --- | --- | --- |
| **LE8 variables** | **Depressive symptom** | |
|  | **AOR (95% CI)^a^** | ***P*-value** |
| **Total** | **0.995 (0.995, 0.996)** | **< 0.001** |
| **Diet** | **0.996 (0.994, 0.997)** | **< 0.001** |
| **PA** | **0.996 (0.995, 0.997)** | **< 0.001** |
| **Nicotine exposure** | **0.993 (0.991, 0.994)** | **< 0.001** |
| **Sleep health** | **0.985 (0.984, 0.987)** | **< 0.001** |
| **BMI** | **0.995 (0.993, 0.996)** | **< 0.001** |
| **Blood lipids (non-HDL-C)** | **0.998 (0.996, 0.999)** | **< 0.05** |
| **Blood glucose** | **0.996 (0.994, 0.998)** | **< 0.001** |
| **Blood pressure** | 1.001 (0.999, 1.003) | 0.412 |
| Footnotes: The total LE8 score was calculated as the mean of the sum of all 8 items of LE8 score and similarly ranged from 0 (if the mean score of all items was 0) to 100 (optimal CVH). Results of AOR (95% CI) and *P*-value presented with bold valued were statistically significant with *P*-value < 0.05 or *P*-value < 0.001. ^a^ For each sub-item of LE8 score: multivariable model was adjusted for age, gender, race/ethnicity, PIR, education level, alcohol consumption, marital status. In the case of total LE8 score, multivariable model was adjusted for age, gender, race/ethnicity, PIR, education level, alcohol consumption, and marital status. | | |
| Abbreviations: AOR, Adjusted odds ratio; BMI, Body mass index; CI, Confidence interval; CVH, Cardiovascular health; LE8, Life’s Essential 8; NHANES, National Health and Nutrition Examination Survey; Non-HDL-C, Non-high-density lipoprotein cholesterol; PA, Physical activity; PIR, Poverty-to-income ratio. | | |

| **Supplementary Table S14** Sensitivity analyses for total and individual LE8 score associated with risk of depressive symptom by calculating the cumulative number of favorable LE8 score and using unfavorable/intermediate score as the reference, NHANES 2005-2018, U.S (n = 25,357) | | | | | | | | | |
| --- | --- | --- | --- | --- | --- | --- | --- | --- | --- |
| **LE8 variables** | **Cases/participants** | **Crude model** | |  | **Model 1** | |  | **Model 2** | |
|  |  | **COR (95% CI)** | ***P*-value** |  | **AOR (95% CI)** | ***P*-value** |  | **AOR (95% CI)** | ***P*-value** |
| **Cumulative number of favorable LE8 score** |  |  |  |  |  |  |  |  |  |
| 0 | 63/302 | Reference | - |  | Reference | - |  | Reference | - |
| 1 | 255/1,603 | **0.718 (0.530,0.983)** | **< 0.05** |  | **0.694 (0.510,0.955)** | **< 0.05** |  | 0.769 (0.561,1.066) | 0.109 |
| 2 | 466/3,704 | **0.546 (0.409,0.738)** | **< 0.001** |  | **0.528 (0.394,0.717)** | **< 0.001** |  | **0.616 (0.456,0.842)** | **< 0.05** |
| 3 | 568/5,798 | **0.412 (0.310,0.555)** | **< 0.001** |  | **0.375 (0.281,0.508)** | **< 0.001** |  | **0.480 (0.357,0.655)** | **< 0.001** |
| 4 | 438/5,840 | **0.308 (0.231,0.416)** | **< 0.001** |  | **0.263 (0.196,0.357)** | **< 0.001** |  | **0.363 (0.268,0.497)** | **< 0.001** |
| 5 | 253/4,277 | **0.239 (0.177,0.326)** | **< 0.001** |  | **0.188 (0.138,0.259)** | **< 0.001** |  | **0.274 (0.200,0.381)** | **< 0.001** |
| ≥ 6 | 151/3,833 | **0.156 (0.113,0.216)** | **< 0.001** |  | **0.108 (0.078,0.152)** | **< 0.001** |  | **0.187 (0.133,0.264)** | **< 0.001** |
| *P* for trend | - | - | **< 0.001** |  | - | **< 0.001** |  | - | **< 0.001** |
| **Diet** |  |  |  |  |  |  |  |  |  |
| Unfavorable/intermediate score | 1,843/19,084 | Reference | - |  | Reference | - |  | Reference | - |
| Favorable score | 351/6,273 | **0.554 (0.492,0.623)** | **< 0.001** |  | **0.532 (0.471,0.599)** | **< 0.001** |  | **0.787 (0.687,0.899)** | **< 0.001** |
| **PA** |  |  |  |  |  |  |  |  |  |
| Unfavorable/intermediate score | 1,043/9,289 | Reference | - |  | Reference | - |  | Reference | - |
| Favorable score | 1,151/16,068 | **0.610 (0.559,0.666)** | **< 0.001** |  | **0.601 (0.549,0.658)** | **< 0.001** |  | **0.732 (0.661,0.810)** | **< 0.001** |
| **Nicotine exposure** |  |  |  |  |  |  |  |  |  |
| Unfavorable/intermediate score | 1,321/11,486 | Reference | - |  | Reference | - |  | Reference | - |
| Favorable score | 873/13,871 | **0.517 (0.473,0.565)** | **< 0.001** |  | **0.443 (0.403,0.486)** | **< 0.001** |  | **0.608 (0.546,0.677)** | **< 0.001** |
| **Sleep health** |  |  |  |  |  |  |  |  |  |
| Unfavorable/intermediate score | 1,260/9,923 | Reference | - |  | Reference | - |  | Reference | - |
| Favorable score | 934/15,434 | **0.443 (0.405,0.484)** | **< 0.001** |  | **0.435 (0.398,0.476)** | **< 0.001** |  | **0.527 (0.477,0.581)** | **< 0.001** |
| **BMI** |  |  |  |  |  |  |  |  |  |
| Unfavorable/intermediate score | 1,686/18,276 | Reference | - |  | Reference | - |  | Reference | - |
| Favorable score | 508/7,081 | **0.760 (0.685,0.843)** | **< 0.001** |  | **0.743 (0.668,0.825)** | **< 0.001** |  | **0.839 (0.743,0.944)** | **< 0.05** |
| **Blood lipids (non–HDL-C)** |  |  |  |  |  |  |  |  |  |
| Unfavorable/intermediate score | 1,364/14,943 | Reference | - |  | Reference | - |  | Reference | - |
| Favorable score | 830/10,414 | **0.862 (0.788,0.943)** | **< 0.001** |  | **0.879 (0.802,0.964)** | **< 0.05** |  | 0.969 (0.875,1.072) | 0.542 |
| **Blood glucose** |  |  |  |  |  |  |  |  |  |
| Unfavorable/intermediate score | 969/9,690 | Reference | - |  | Reference | - |  | Reference | - |
| Favorable score | 1,225/15,667 | **0.763 (0.699,0.834)** | **< 0.001** |  | **0.675 (0.613,0.744)** | **< 0.001** |  | **0.863 (0.773,0.964)** | **< 0.05** |
| **Blood pressure** |  |  |  |  |  |  |  |  |  |
| Unfavorable/intermediate score | 1,214/14,450 | Reference | - |  | Reference | - |  | Reference | - |
| Favorable score | 980/10,907 | 1.076 (0.985,1.175) | 0.102 |  | 0.966 (0.879,1.062) | 0.477 |  | **1.116 (1.006,1.239)** | **< 0.05** |
| Footnotes: Cumulative number of favorable LE8 score was calculated by accumulating the 8 dichotomized items of LE8 score, and thus the score range from 0 to 8. Score for 8 items of LE8 was categorized into two groups (unfavorable/intermediate score and favorable score, with scores of 0 and 1 to represent an unfavorable/intermediate score level and favorable score level, respectively) for sensitivity analyses. For Cumulative number of favorable LE8 score: Crude model was unadjusted. Model 1 was adjusted for age, gender, and race/ethnicity. Model 2 was adjusted for age, gender, race/ethnicity, PIR, education level, alcohol consumption, marital status. For each sub-item of LE8 score: Model 2 was adjusted for age, gender, race/ethnicity, PIR, education level, alcohol consumption, marital status. Results of COR (95% CI), AOR (95% CI), *P* for trend, and *P*-value presented with bold valued were statistically significant with *P*-value < 0.05 or *P*-value < 0.001. | | | | | | | | | |
| Abbreviations: AOR, Adjusted odds ratio; BMI, Body mass index; CI, Confidence interval; COR, Crude odds ratio; LE8, Life’s Essential 8; NHANES, National Health and Nutrition Examination Survey; Non-HDL-C, Non-high-density lipoprotein cholesterol; PA, Physical activity; PIR, Poverty-to-income ratio. | | | | | | | | | |
